# The hepatocyte-specifically expressed lnc-HSER alleviates hepatic fibrosis by inhibiting hepatocyte apoptosis and epithelial-mesenchymal transition

**DOI:** 10.7150/thno.36942

**Published:** 2019-10-12

**Authors:** Kun Zhang, Mengxia Zhang, Qingbin Yao, Xiaohui Han, Yanmian Zhao, Lina Zheng, Guantong Li, Qi Liu, Yanan Chang, Peijun Zhang, Hongmei Cui, Zhemin Shi, Ting Chen, Zhi Yao, Tao Han, Wei Hong

**Affiliations:** 1 Department of Histology and Embryology, Key Laboratory of Immune Microenvironment and Disease of Ministry of Education, Tianjin Key Laboratory of Cellular and Molecular Immunology, School of Basic Medical Sciences, Tianjin Medical University, Tianjin, China; 2 The Third Central Clinical College of Tianjin Medical University, Department of Hepatology and Gastroenterology, Tianjin Third Central Hospital, Tianjin Key Laboratory of Artificial Cells, Artificial Cell Engineering Technology Research Center of Public Health Ministry, Tianjin, China; 3 Department of Pathology, Institute of Hematology and Blood Diseases Hospital, Chinese Academy of Medical Sciences and Peking Union Medical College, Tianjin, China; 4 Tianjin Central Hospital of Gynecology Obstetrics, Tianjin, China; 5 Department of Immunology, Key Laboratory of Immune Microenvironment and Disease of Ministry of Education, Tianjin Key Laboratory of Cellular and Molecular Immunology, School of Basic Medical Sciences, Tianjin Medical University, Tianjin, China

**Keywords:** lncRNA, liver fibrosis, hepatocyte, C5AR1, Notch

## Abstract

Liver fibrosis leading to cirrhosis is one of the major health burdens worldwide with currently limited therapeutic options available. Long noncoding RNAs (lncRNAs) play important roles in various biological and pathological processes in a cell- or tissue-specific manner. However, there is still an important gap in the understanding of the role of hepatocyte-specific lncRNAs in liver fibrosis.

**Methods:** The expressions of lnc-Hser in human and mice fibrotic livers as well as primary hepatocytes (HCs) of mice developing liver fibrosis were determined by real-time RT-PCR. The roles and mechanisms of lnc-Hser in HCs and liver fibrosis were determined *in vitro* and *in vivo*.

**Results:** In this study, we have identified a hepatocyte-specifically expressed lnc-Hser, which was reduced in human and mice fibrotic livers as well as primary HCs of mice developing liver fibrosis. We have shown that silencing lnc-Hser aggravated liver fibrosis both* in vitro* and *in vivo* through inducing the epithelial-mesenchymal transition (EMT) and the apoptosis of HCs. In addition, knockdown of lnc-Hser promoted hepatic stellate cells (HSCs) activation through the signals derived from injured HCs. Mechanistically, we have revealed that lnc-Hser inhibited HCs apoptosis via the C5AR1-Hippo-YAP pathway and suppressed HCs EMT via the Notch signaling.

**Conclusions:** Our work has identified a hepatocyte-specific lnc-HSER that regulates liver fibrosis, providing a proof that this molecule is a novel biomarker for damaged HCs and a potential target for anti-fibrotic therapy.

## Introduction

Liver fibrosis is a common pathological consequence of a sustained wound healing response to chronic liver injury, including viral hepatitis, alcohol abuse and autoimmune disease, characterized by abnormal deposition of extracellular matrix (ECM) and diminished hepatocytes (HCs) regeneration that is responsible for compromised liver function accounting for death 1, 2. Progressive liver fibrosis leads to cirrhosis and its associated complications include portal hypertension, liver failure, and the high risk of incident hepatocellular carcinoma 3, 4. Current models of chronic liver injury of various etiologies emphasize HCs apoptosis as a critical initiator of fibrosis by triggering HSCs to trans-differentiate into myofibroblasts with an up-regulation of pro-fibrogenic cytokines such as TGFβ, and an increased production of ECM compounds 5. In addition, HCs EMT is also an important cellular event propagating liver fibrosis progression 6. Therefore, developing a new therapeutic approach to protect HCs from apoptosis and EMT is needed.

Many molecules and signalings have been documented to initiate HCs apoptosis and EMT in liver fibrosis, including TGFβ1, Notch and Hippo pathways. TGFβ1, a known pro-apoptotic cytokine in mature HCs, is capable of mediating phenotypic changes and plasticity in the process of EMT, resulting in ECM deposition 6. The Notch signaling network is an evolutionarily conserved intercellular signaling that regulates a range of cellular functions including proliferation, apoptosis, cell fate and differentiation 7. Triggering Notch receptors (Notch1-4) activates proteolytic cleavages catalyzed by γ-secretase, generating the Notch intracellular domain (NICD), which enters nucleus to activate transcription of canonical Notch targets including the Hairy enhancer of split (Hes) and Hes-related (Hey) family genes through the transcription factor RBPJ. Of note, increasing evidences suggest that Notch signaling also contributes to the progression of liver fibrosis via inducing the EMT 8. Moreover, it is well established that isolated HCs in culture rapidly lose their epithelial and functional properties and spontaneously undergo a de-differentiation process towards a mesenchymal cell type. This process, which exerts features of the EMT, is blocked in the presence of several inhibitors including Notch inhibitor 9. The Hippo pathway is an important modulator involved in the regulatory process of cell proliferation, differentiation and death. Central to the Hippo pathway is a kinase cascade wherein MST (the mammalian orthologue of the Drosophila Hippo) phosphorylates and activates the adaptor protein Mob and the protein kinase LATS. Activated LATS phosphorylates the transcriptional co-activator YAP/TAZ, binding to 14.3.3 proteins in the cytoplasm, which accelerates the sequestration and degradation of YAP/TAZ. Unphosphorylated YAP translocates into the nucleus, interacts with a number of transcriptional factors to mediate the transcription of genes that control cell proliferation and apoptosis 10. Additionally, G-protein-coupled receptors (GPCRs), the largest family of cell surface receptors, have been reported to function upstream of the Hippo pathway through Rho GTPase and cytoskeleton remodeling 11. Interestingly, several lines of evidence indicate that the Hippo/YAP signaling plays a critical role in liver fibrosis. Some studies including our preliminary study found that YAP is increased and undergoes a nuclear localization at an early stage during the activation of HSCs, indicating that YAP may function at the earliest stage of HSCs activation 12, 13. However, the role of YAP in HCs during liver fibrogenesis is still unclear. It has been reported that YAP promotes HCs death and subsequently leads to liver fibrosis 14, while others revealed that YAP protects HCs from death 15, 16. Hence, further understanding of the cellular and molecular mechanism of liver fibrosis may lead to the development of more effective treatments.

Long noncoding RNAs (lncRNAs) represent a group of transcripts that are above 200 nucleotides in length but without protein coding potential 17, 18. Emerging evidences have demonstrated that lncRNAs are expressed in a cell- or tissue-specific manner and involved in a variety of biological and pathological processes including organ fibrosis 19-25. We and others have revealed that lncRNAs were implicated in HSCs activation and thereby regulate liver fibrosis. For example, H19 plays a critical role in the progression of cholestatic liver fibrosis either by promoting HSCs differentiation and activation or by preventing ZEB1-mediated inhibition of epithelial cell adhesion molecule 23, 26. Our group have recently demonstrated that a liver-enriched lncRNA LFAR1 promoted HSCs activation and subsequently led to liver fibrosis 22, and a nuclear-retained lncRNA SCARNA10 functioned as a novel positive regulator of TGFβ signaling in liver fibrogenesis by inhibiting the binding of PRC2 to the promoters of the genes associated with ECM and TGFβ pathway 27. However, there is still an important gap in understanding the role of cell-specific lncRNAs that mainly affect HCs during liver fibrogenesis.

In the present study, we have identified a HC-specifically expressed lnc-Hser, which was reduced in human and mice fibrotic livers as well as primary HCs of mice developing liver fibrosis. We have shown that silencing lnc-Hser aggravated liver fibrosis both* in vitro* and *in vivo* through inducing the EMT and the apoptosis of HCs. In addition, knockdown of lnc-Hser promoted HSCs activation through the signals derived from damaged HCs. we have also revealed that lnc-Hser inhibited HCs apoptosis via the C5AR1-Hippo-YAP pathway and suppressed HCs EMT through the Notch signaling. All these data suggest that lnc-HSER is a novel biomarker for damaged HCs and a potential target for anti-fibrotic therapy.

## Materials and methods

### Cell culture and antibodies

The non-tumorigenic mouse hepatocyte cell line AML12 was maintained in Dulbecco's modified Eagle's medium (DMEM, Invitrogen, Camarillo, CA) supplemented with 10% fetal bovine serum (FBS, Gibco, Gaithersburg, MD, USA), 1 × insulin-transferrin-sodium selenite media supplement (ITS; Sigma-Aldrich), dexamethasone (40 ng/ml), penicillin (100 U/ml) and streptomycin (100 μg/ml). The human hepatocyte cell line L02 and HEK293T were cultured in DMEM supplemented with 10% FBS, penicillin (100 U/ml) and streptomycin (100 μg/ml). All cells were cultured at 37°C in an atmosphere containing 5% CO_2_. For co-culture experiment, lnc-Hser-silenced and lnc-Hser-overexpressed AML12 cells and the controls were washed with PBS after 24 - 48h of lentivirus infection. The cells were subsequently incubated in DMEM supplemented with 10% FBS for 48h and the supernatants were centrifuged at 1100 rpm for 5 min and mixed with DMEM containing 10% FBS at 1:1 ratio for preparing conditioned medium (CM). Cells were treated with the C5AR1 inhibitor PMX 205 (Med Chem Express, USA) or γ-secretase inhibitor RO4929097 (Med Chem Express, USA) for 24 hours at concentrations of 5 μM. The antibodies were α-SMA (rabbit polyclonal, Abcam, ab5694), Collagen1 (rabbit polyclonal, Abcam, ab34710; Millipore, #234167), TGFβ (rabbit polyclonal, Abcam, ab66043), MMP2 (rabbit monoclonal, Abcam, ab92536), TIMP1 (mouse monoclonal, Santa Cruz,sc-21734), Notch2 (rabbit monoclonal, Cell Signaling Technology, #5732), Notch3 (rabbit polyclonal, Abcam, ab23426), Hes1 (rabbit polyclonal, Abcam, ab71559), phospho-YAP (Ser-127) (rabbit monoclonal, Cell Signaling Technology, #13619), total YAP/TAZ (rabbit monoclonal, Cell Signaling Technology, #8418), phospho-MST1/2 (rabbit monoclonal, Cell Signaling Technology, #49332), total MST1 (rabbit monoclonal, Cell Signaling Technology, #3682), phospho-LATS (Ser-909) (rabbit polyclonal, Cell Signaling Technology, #9157), total LATS1 (rabbit monoclonal, Cell Signaling Technology, #3477), C5AR1 (rabbit monoclonal, Proteintech, #21316-1-AP), Ki67 (rabbit monoclonal, Abcam, ab16667), Cleaved Caspase3 (rabbit polyclonal, Cell Signaling Technology, #9661), Caspase3 (rabbit monoclonal, Cell Signaling Technology, #9662), BAX (rabbit polyclonal, Abcam, ab32503), N-Cadherin (rabbit monoclonal, Cell Signaling Technology, #13116; Mouse monoclonal, Abcam, ab98952), E-cadherin (rabbit monoclonal, Cell Signaling Technology, #3195), β-Catenin (rabbit monoclonal, Cell Signaling Technology, #8480), Vimentin (rabbit monoclonal, Cell Signaling Technology, #5741), Snail (rabbit monoclonal, Cell Signaling Technology, #3879), rabbit IgG (Millipore, PP64B), goat anti rabbit IgG (Invitrogen, Alexa Fluor 488/594), goat anti mouse IgG (Invitrogen, Alexa Fluor 594).

### Construction of plasmids

gRNA design was based on CRISPR design (http://crispr.mit.edu/) or CHOPCHOP (https://chopchop.rc.fas.harvard.edu/) and cloned into lenti-CRISPRv2. Oligos encoding shRNA specific for lnc-Hser and the negative control shRNA were ligated into pSUPER.retro.puro, and the fragment containing the H1 promoter and hairpin sequences was subcloned into the lentiviral shuttle pCCL.PPT.hPGK.GFP.Wpre (lnc-Hser-shRNA and Negative Control (NC)). The full-length lnc-Hser cDNA was sequentially amplified by PCR and ligated into the lentiviral shuttle pCCL.PPT.hPGK.IRES.eGFP/pre to generate the over-expression plasmid (LV-lnc-Hser and the empty plasmid as the LV-Control). These plasmids were used to produce lentivirus in HEK-293T cells with the packaging plasmids pMD2.BSBG, pMDLg/pRRE and pRSV-REV. Infectious lentiviruses were harvested at 36 h and 60 h after transfection and filtered through 0.45 μm PVDF filters. Recombinant lentiviruses used *in vivo* were concentrated 100-fold by ultracentrifugation (2 h at 120,000 g). The virus-containing pellet was dissolved in PBS and injected in mice within 48 h. The primer sets used are shown in Table S1.

### Animals *in vivo* study

Animal protocols were approved by Tianjin Medical University Animal Care and Use Committee. The methods were carried out in accordance with the approved guidelines. All Balb/c mice aged at 8 weeks were obtained from Institute of Laboratory Animal Sciences, CAMS & PUMC (Beijing, China). For bile duct ligation (BDL) -induced mouse liver fibrosis model, mice were treated with sham operation or BDL operation for 3 to 21 days and all of mice were sacrificed under anesthesia with 3% sodium pentobarbital (45 mg/kg, ip). For carbon tetrachloride (CCl_4_)-induced mouse liver fibrosis model, forty Balb/c mice were randomly divided into four groups: Mice were treated with olive oil in combination with injection of lenti-NC (NC; n = 10), CCl_4_ in combination with injection of lenti-NC (NC + CCl_4_, n = 10), oil in combination with injection of lenti-lnc-Hser-shRNA (lnc-Hser-shRNA, n = 10) and CCl_4_ in combination with injection of lenti-lnc-Hser-shRNA (lnc-Hser-shRNA + CCl_4_, n = 10). The lentivirus was injected via the tail vein 2 weeks after the first injection of CCl_4_ (1 × 10^9^ pfu/mouse). Mice in NC + CCl_4_ group and lnc-Hser-shRNA + CCl_4_ group were administered 5% CCl_4_ (v/v) dissolved in olive oil (0.2 ml/kg body weight) twice per week for additional 4 weeks via intraperitoneal (ip) injection after the lentivirus was injected. NC and lnc-Hser-shRNA group animals were injected with an equivalent volume of olive oil. After treatment with CCl_4_ for 6 weeks, all of mice were sacrificed under anesthesia with 3% sodium pentobarbital (45 mg/kg, ip). Liver specimens and serums were obtained for analyses.

### Histology and immunohistochemistry

The specimens were sequentially fixed in 10% formalin for two days, transferred to ethanol of different concentration and embedded in paraffin in preparation for histopathological analysis. Thin sections (5 μm) were stained with H*&*E, Sirius red and Masson's trichrome staining for histopathological study. According to the above results, three sections were chosen from each group for immunohistochemical analysis. Briefly, sections prepared on slides were firstly submitted to antigen retrieval by incubation in citrate buffer (pH 6.0) for 5 min at 108 °C and pretreated with 3% H_2_O_2_ for 15 min at room temperature followed by washing with PBS. Slides were subsequently incubated in normal goat serum for 20 min to block the nonspecific immunoreactivity. Next, the slides were treated with primary antibody α-SMA (1:50), Col1α1 (1:1000) or TGFβ (1:50) overnight at 4°C. In addition, tissue sections were processed omitting the primary antibody as the negative control. The slides were incubated with secondary antibody (1:500) (HRP-conjugated anti-rabbit IgG) and the reaction products were visualized using diaminobenzidine (DAB) and monitored by microscopy.

### ELISA and liver enzyme measurement

The TGFβ level in cell culture media and the alanine aminotransferase (ALT) and aspartate aminotransferase (AST) levels in the serum were assessed using commercial assay kits (Nanjing Jiancheng Corp., Nanjing, China) according to the manufacturer's protocols.

### Hydroxyproline assay

Total collagen content was tested by measuring the amount of hydroxyproline in liver tissue using commercially available hydroxyproline detection kits (Nanjing Jiancheng Corp., Nanjing, China) according to the manufacturer's instructions.

### Isolation and culture of primary HCs, HSCs, liver sinusoidal endothelial cell (LSECs) and Kupffer cell (KCs)

Primary mouse HCs, HSCs, LSECs and KCs were isolated by pronase/collagenase perfusion digestion followed by subsequent density gradient centrifugation, as previously described 22. Magnetic cells sorting (MACS) and/or selective adhesion was further employed to isolate LSECs (CD146) and KCs (F4/80). In brief, primary HCs were isolated from the 6-week-old Balb/c mice by *in situ* perfusion with 30 ml SC1 solution and 30 ml 0.05% Collagenase IV solution sequentially. HCs were then pelleted by centrifugation 50 g for 4 minutes three times. Cell viability was determined by the trypan blue exclusion method.

### TUNEL assay

For TUNEL staining, we used an in-situ cell detection kit (Roche) according to the manufacturer's protocol. After dewaxing and rehydration, we pretreated tissue sections with 3% H_2_O_2_ and subsequent proteinase K permeation. Pretreatment with DNase I served as a positive control and TUNEL reaction mixture lacking terminal transferase (TdT) as a negative control. Samples were analyzed by light microscopy (×400 magnification).

### Nuclear-cytoplasmic fractionation

Cytoplasmic and nuclear RNA isolation were performed with PARIS™ Kit (Invitrogen, Grand Island, NY, USA) following the manufacturer's instruction, as previously described 22.

### 5' and 3' rapid amplification of cDNA ends (RACE)

We used the 5'-RACE and 3'-RACE analyses to determine the transcriptional initiation and termination sites of lnc-Hser using a SMARTer™ RACE cDNA Amplification Kit (Clontech, Palo Alto, CA) according to the manufacturer's instructions. In brief, RNA was isolated from primary HCs and 3'- and 5'-RACE-Ready cDNA were synthesized using SMART Scribe Reverse Transcriptase. The obtained band was gel purified and cloned with the lineareized pRACE vector. The obtained band was then sequenced. The gene-specific primers used for the PCR of the RACE analysis are provided in Table S1.

### Apoptosis assay

The apoptosis of cells with different treatment was analyzed using a FITC Annexin V Apoptosis Detection Kit I (BD Biosciences) according to the manufacturer's instruction, as previously described 22.

### Quantitative real-time polymerase chain reaction

Total RNA was extracted from livers or cells with Trizol reagent (Takara, Dalian, China) and nuclear and cytoplasmic RNA were prepared using PARIS™ Kit (Invitrogen, Grand Island, NY, USA). All RNAs were digested with DNase I (Takara, Dalian, China). Briefly, the first-strand cDNA was synthesized using AMV Reverse Transcriptase (Thermo Fisher Scientific, Basingstoke, UK). For real-time PCR, all reactions were performed in triplicate with SYBR Green master mix (Takara, Dalian, China) according to the manufacturer's instructions. The expression level of housekeeping gene GAPDH was used to normalize the expression level of the genes-of-interest. The sequences of primers for real-time PCR are listed in Table S1.

### Western blot analysis

Immunoblotting analysis was performed as described previously 28. The antibodies against α-SMA (1:1000), Collagen1 (1:1000), TGFβ (1:1000), MMP2 (1:1000), TIMP1(1:1000), Notch2 (1:1000), Notch3 (1:1000), Hes1 (1:1000), phospho-YAP (Ser-127) (1:1000), total YAP/TAZ (1:1000), phospho-MST1/2 (1:1000), total MST1 (1:1000), phospho-LATS (Ser-909) (1:1000), total LATS1 (1:1000), C5AR1 (1:1000), Caspase3 (1:1000), BAX (1:1000), N-Cadherin (1:1000), E-cadherin (1:1000), β-Catenin (1:1000), Vimentin (1:1000), Snail (1:1000) and GAPDH (1:8000) were diluted in TBS containing 5% milk and 0.1% Tween 20. Signal was detected using the chemiluminescence (ECL) system (Millipore).

### Confocal microscopy

Primary HSCs, HCs and AML12 cells were re-plated on poly-lysine pre-coated glass coverslips, and were sequentially fixed with 4% paraformaldehyde in PBS overnight at 4 °C, permeabilized with 1% Triton X-100 in PBS for 0-60 min and blocked using 5% bovine serum albumin (BSA) in TBST with 0.1% Tween-20 for 30-60 min at room temperature. Next, the cells were incubated with primary antibodies against α-SMA (1:300), Col1α1 (1:500), Cleaved Caspase3 (1:400), N-Cadherin (Mouse monoclonal; 1:200) and Ki67 (1:250), overnight at 4 °C and an irrelevant isotype rabbit IgG was used as a negative control. Cells were then incubated with Alexa Fluor 488 (1:400 for Cleaved Caspase3) or Alexa Fluor 594 (1:400 for α-SMA, Col1α1, N-Cadherin and Ki67) in PBS away from light for 1 h at room temperature and the nuclei were stained with DAPI (5 μg/ml). Finally, the slides were washed with PBS and the coverslips were mounted with an anti-fade Mounting Medium (P0126, Beyotime, Shanghai, China). The immunofluorescence was then visualized by a confocal microscope (LSM 700) or a fluorescence microscope.

### Study population

In total, 28 human fibrotic livers and 6 human healthy livers from patients with hepatic haemangioma were obtained from surgical resections without preoperative treatment at Tianjin Third Central Hospital (Tianjin, China). For histological scoring of liver fibrosis, we stained paraffin embedded 5 μm liver sections with Sirius red, H&E and Masson's trichrome staining. Hepatic fibrosis was scored (stages F0-F4) according to the METAVIR fibrosis staging system by three hepatopathologists blinded to the study protocol and stratified as normal liver (F0), mild fibrosis (F1-F2) or advanced fibrosis (F3-F4). All subjects were of the same ethnicity. Clinical and pathological characteristics including age, gender, ALT, AST and etiologies were recorded and summarized in Table S2. The study has been approved by the local Ethical Committee of Tianjin Third Central Hospital (Tianjin, China). Written informed consent was obtained from each patient according to the policies of the committee. The study methodologies were conformed to the standards set by the Declaration of Helsinki.

### Statistical analysis

Data were expressed as mean ± SD. All the statistical analyses were performed with the SPSS 13.0 (IBM, Armonk, NY, USA). Statistical analyses were performed using either Student's *t*-test (two-group comparison) or one-way analysis of variance (more than two groups) followed by* post hoc* comparison, and differences with *p*<0.05 were considered significantly.

## Results

### lncRNA-Hser is specifically expressed in hepatocyte of normal liver

**Figure 1:**
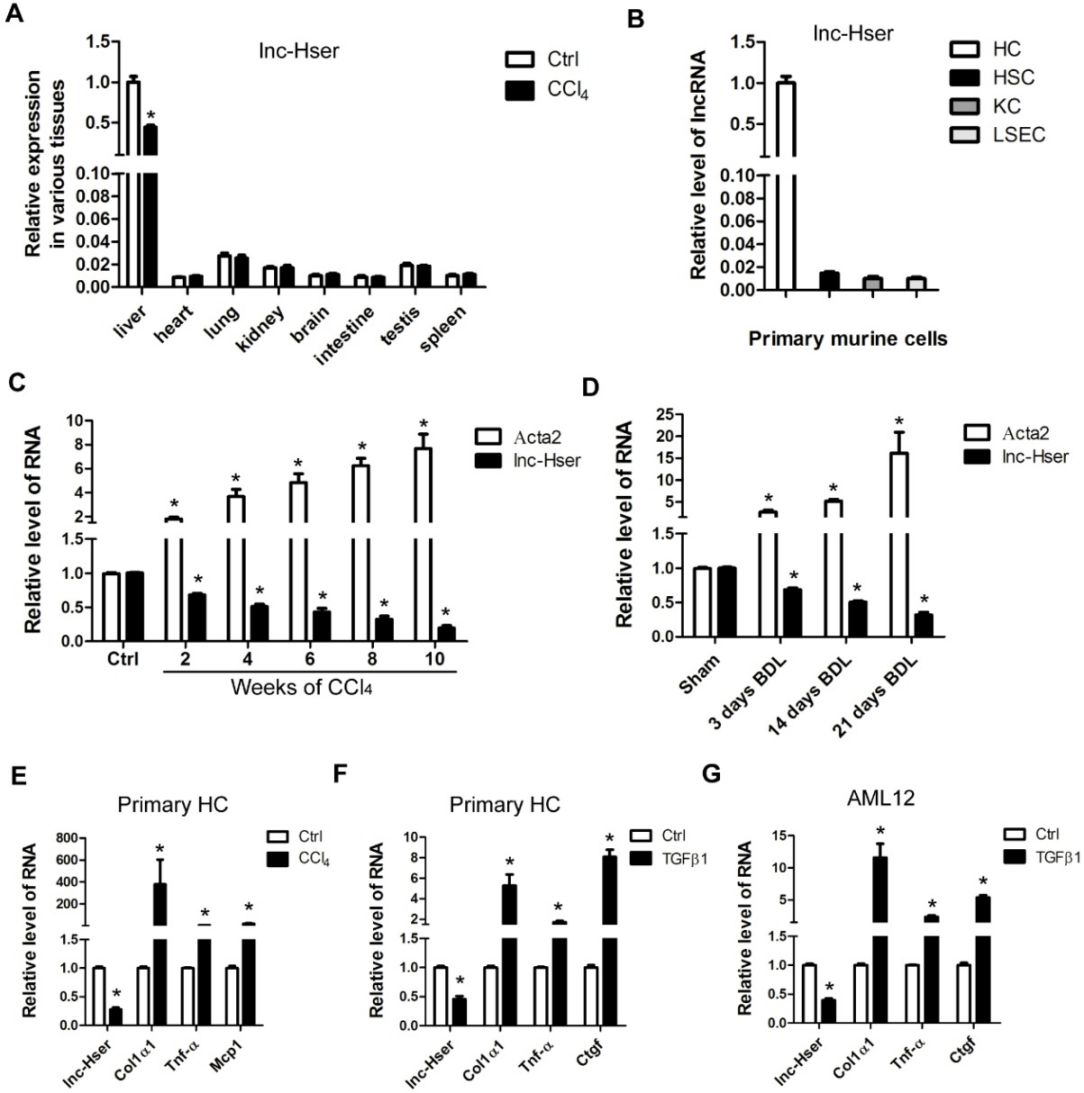
** lnc-Hser is specifically expressed in HCs and down-regulated in fibrotic HCs and livers.** (A) qRT-PCR analysis of *lnc-Hser* in various tissues from normal and fibrotic mice. (B) HCs, HSCs, KCs and LSECs were isolated from livers of Balb/c mice, and *lnc-Hser* expression was analyzed by qRT-PCR. (C, D) qRT-PCR analysis of *lnc-Hser* and *Acta2 (α-SMA)* in livers from mice treated with CCl_4_ (C) or undergone BDL (D) for indicated times. (E) Primary HCs were isolated from livers of mice treated with CCl_4_ or oil for 6 weeks, and the transcript of *lnc-Hser, Col1α1, Tnf-α* and *Mcp1* was determined by qRT-PCR. (F, G) Primary HCs (F) and AML12 cells (G) were stimulated with TGFβ for 24 h and *lnc-Hser, Col1α1, Tnf-α* and *Ctgf* was determined by qRT-PCR. The data are expressed as the mean ± SD for at least triplicate experiments, *p< 0.05.

We have previously identified systemic variations in the expression of lncRNAs between mouse fibrotic and normal livers using a microarray analysis 22. From that study, we noted that lncRNA ENSMUST00000154817 was significantly down-regulated in liver fibrosis according to the microarray data. (The microarray data discussed in that article have been deposited in NCBI Gene Expression Omnibus and are accessible through GEO Series accession number GSE80601). To explore the role of this molecule in liver fibrosis, we have firstly examined its expression patterns in various tissues of normal and fibrotic mice. As shown in Figure 1A, normal livers express highest level ENSMUST00000154817, which is down-regulated in fibrotic livers. In addition, we have isolated four types of primary cells including HCs, HSCs, LSECs and KCs from livers of healthy mice, and found ENSMUST00000154817 was mainly expressed in primary HCs rather than HSCs, LSECs and KCs (Figure 1B). These data demonstrated ENSMUST00000154817 was a HCs-specifically expressed lncRNA, and thus was named as lnc-Hser (**h**epatocyte **s**pecific **e**xpressed lnc**R**NA). We have subsequently performed 5'- and 3'- RACE assay with primary HCs and found lnc-Hser was a 588-nucleotide transcript with poly (A) tail, inconsistent with the ensemble database (Figure S1A). The discrepancy may be due to the existence of different isoforms in various tissues. Cell fractionation followed by qRT-PCR showed that lnc-Hser was mainly located in the nuclei of primary HCs and AML12 cells (Figure S1B, C). As expected, the full-length transcript of lnc-Hser has no protein coding potential according to the coding potential calculator 2 (CPC2) and coding potential assessment tool (CPAT). The expression of lnc-Hser was then measured in total RNA extracts from fibrotic livers of mice treated with CCl_4_ or BDL for various time periods, and the results showed that the transcript of lnc-Hser was significantly down-regulated with persistent injury, correlating with gradual enhancement of α-SMA (Acta2) (Figure 1C, D). We have also isolated primary HCs from livers of mice treated with CCl_4_ for 6 weeks and found lnc-Hser exhibited a clear down-regulation compared with control, correlating with an up-regulation of Col1α1, Tnf-α and Mcp1 (Figure 1E). Notably, stimulation of primary HCs and AML12 cells with recombinant TGFβ1 resulted in a significantly reduced level of lnc-Hser (Figure 1F, G). Collectively, these data demonstrate that lnc-Hser is decreased in the livers and HCs of fibrotic mice, leading us to explore the possibility that lnc-Hser may be a regulator to the progression of liver fibrosis.

### Knockdown of lnc-Hser facilitates the progression of liver fibrosis

**Figure 2:**
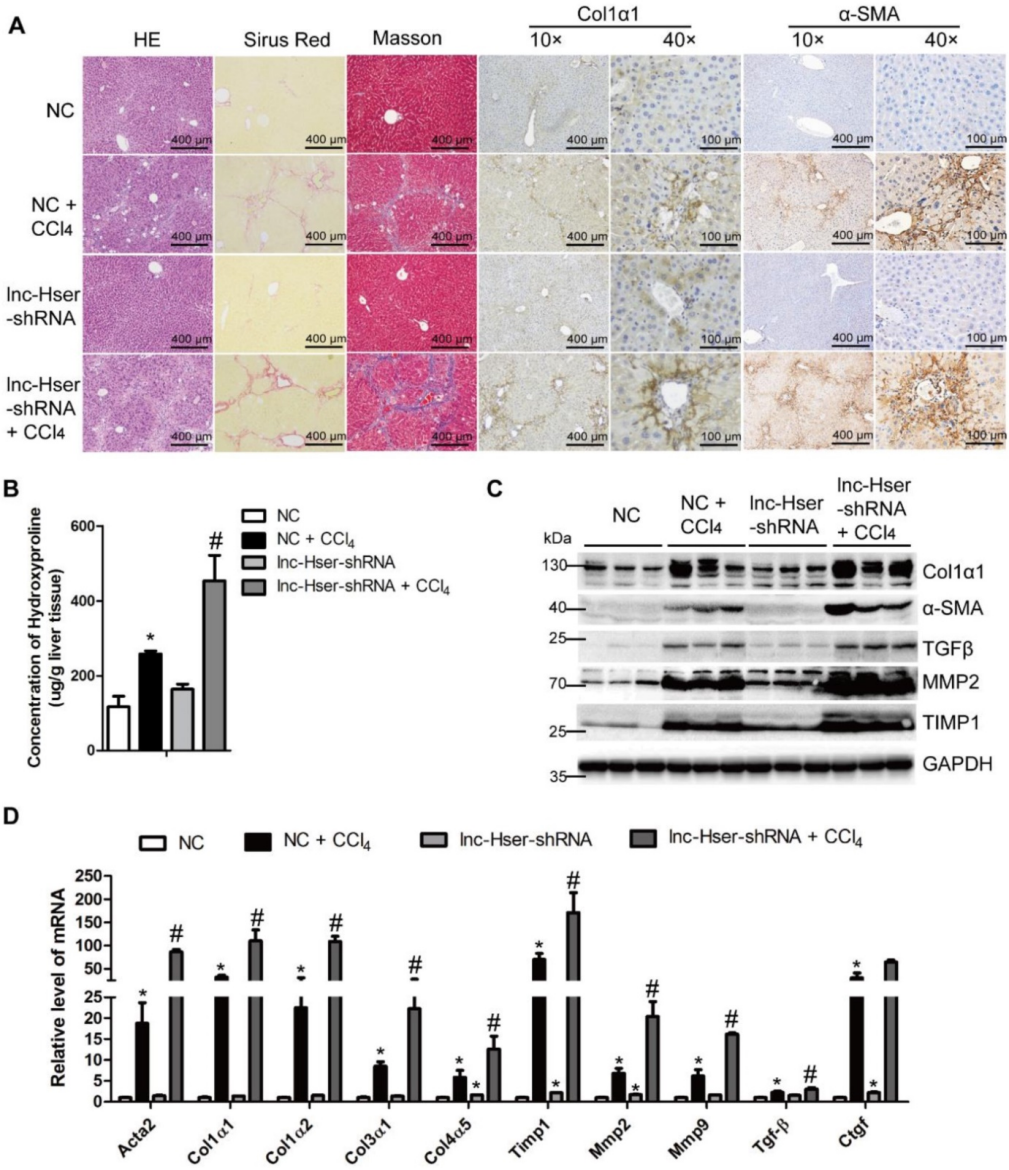
** Knockdown of lnc-Hser aggravates CCl_4_-induced liver fibrosis* in vivo*.** Mice were treated with oil in combination with injection of lenti-NC (Negative Control, n = 10), or CCl_4_ in combination with injection of lenti-NC (NC + CCl_4_, n = 10), or oil in combination with injection of lenti-lnc-Hser-shRNA (lnc-Hser-shRNA, n = 10), or CCl_4_ in combination with injection of lenti-lnc-Hser-shRNA (lnc-Hser-shRNA + CCl_4_, n = 10). (A) Liver fibrosis was evaluated by stainings of H&E, Sirius red, Masson's trichrome, IHC for Collagen1 and α-SMA; scale bar = 400 μm for 10× and 100 μm for 40×. (B) Quantification of hepatic hydroxyproline content. The data are expressed as hydroxyproline (μg)/liver wet weight (g). (C) The protein level of α-SMA, Collagen1, TGFβ, MMP2 and TIMP1 was determined by western blot. GAPDH was used as an internal control. (D) The mRNA level of the predominant fibrotic genes, including *α-SMA, Col1α1, Col1α2, Col3α1, Col4α5, Tgfβ, Timp1, Mmp2, Mmp9* and *Ctgf*, was determined by qRT-PCR. The data are expressed as the mean ± SD for at least triplicate experiments, *p<0.05 stands for NC + CCl_4_ or lnc-Hser-shRNA vs NC. #*p*<0.05 stands for lnc-Hser-shRNA + CCl_4_ vs NC + CCl_4_.

To elucidate the *in vivo* function of lnc-Hser in hepatic fibrogenesis, we generated lnc-Hser down-expressed mouse models via tail vein injection of lenti-lnc-Hser-shRNA (lnc-Hser-shRNA) or lenti-negative control virus (NC) 2 weeks after the first injection of CCl_4_. After 6 weeks of CCl_4_ treatment, lnc-Hser silencing was confirmed by qRT-PCR in whole liver extracts (Figure S2A), and the CCl_4_ group mice infected with NC (NC-CCl_4_) developed liver fibrosis. However, administration of lnc-Hser-shRNA in CCl_4_ group (lnc-Hser-shRNA-CCl_4_) developed more severe phenotypes of fibrosis as demonstrated by both the staining of H&E, Sirius red, Masson's trichrome and the IHC for α-SMA, Collagen1 and TGFβ (Figure 2A and Figure S2B). The content of liver hydroxyproline in lnc-Hser-shRNA-CCl_4_ mice was also significantly higher in comparison with the NC-CCl_4_ mice (Figure 2B). Moreover, the expression of the predominant fibrotic genes including α-SMA, Col1α1, TGFβ, TIMP1, MMP2 and MMP9 was noticeably increased by CCl_4_ and was further enhanced in lnc-Hser-shRNA-CCl_4_ mice (Figure 2C, D). Notably, increased TUNEL positive HCs and serum level of ALT and AST were observed in lnc-Hser-shRNA-CCl_4_ versus NC-CCl_4_ mice, suggesting lnc-Hser silencing worsened CCl_4_-induced HCs injury (Figure S2B, C and Table S3). Consistent with these observations, knockdown of lnc-Hser exacerbated CCl_4_-induced HCs apoptosis assessed by IHC-Frozen, western blot and qRT-PCR (Figure S2D-F). In addition, the expression of the genes related to hepatic inflammation including Tnf-α, Il-1β, Mcp1 and Il-6, the mesenchymal markers N-Cadherin, β-Catenin, Vimentin and Snail1 was dramatically enhanced in lnc-Hser-shRNA-CCl_4_ versus NC-CCl_4_ mice (Figure S2D-F). Taken together, our results suggest that silencing lnc-Hser aggravates CCl_4_-induced liver fibrosis* in vivo* through inducing the EMT and the apoptosis of HCs.

### lnc-Hser ameliorates apoptosis and EMT of HCs* in vitro*

**Figure 3:**
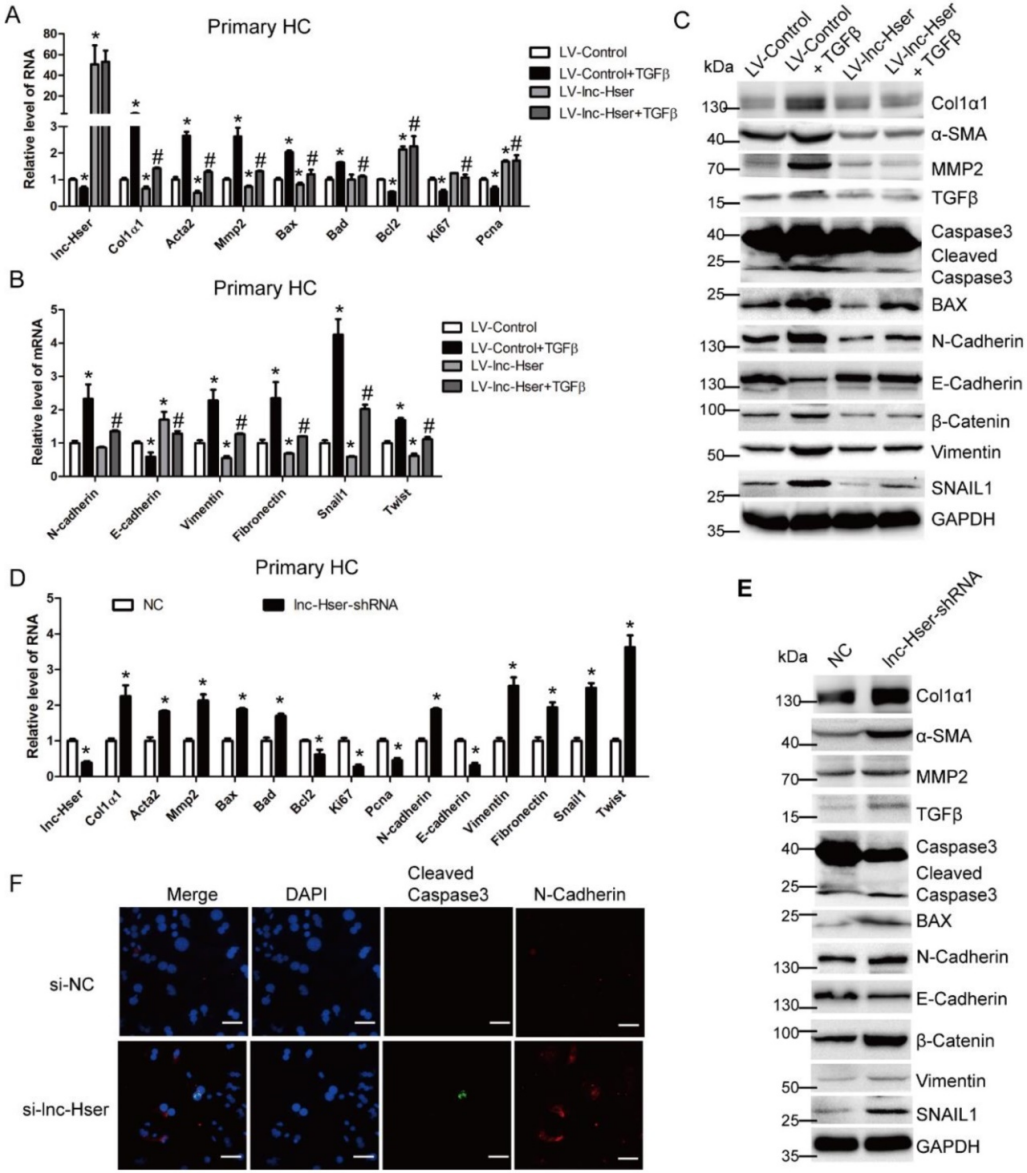
** lnc-Hser ameliorates apoptosis and EMT of HCs* in vitro.***(A-C) Primary HCs were infected with LV-lnc-Hser for 72 h and further treated with 10 ng/ml TGFβ for additional 24 h. The expression of *lnc-Hser,* pro-fibrogenic genes (*Acta2, Col1α1* and* Mmp2*), apoptosis and proliferation -related genes (*Bax, Bad, Bcl2, Ki67* and *Pcna*) and EMT-related genes (*N-Cadherin, E-cadherin, Vimentin, Fibronectin, Twist* and *Snail1*) was detected by qRT-PCR (A, B). The protein level of α-SMA, Col1α1, MMP2, total and cleaved Caspase3, BAX, N-Cadherin, E-cadherin, Vimentin, β-Catenin and SNAIL1 was detected by western blot. GAPDH was used as an internal control (C). (D, E) The expression of lnc-Hser, pro-fibrogenic genes, apoptosis-related genes and EMT-related genes was detected in primary HCs infected with lenti-lnc-Hser-shRNA or lenti-NC by qRT-PCR (D) and western blot. GAPDH was used as an internal control (E). (F) Primary HCs were transfected with siRNA-lnc-Hser or si-NC for 36 h, the expression and location of cleaved Caspase3 and N-Cadherin was determined by confocal microscopy. DAPI stained nuclei blue; scale bar = 50 μm. The data are expressed as the mean ± SD for at least triplicate experiments. *p<0.05 stands for vs LV-Control or NC. #*p*<0.05 stands for vs LV-Control + TGFβ or NC+ CCl_4_.

TGFβ1, a known pro-apoptotic cytokine in mature HCs, is also a major inducer of EMT 6. To investigate whether lnc-Hser is required for the apoptosis and EMT of HCs* in vitro*, we firstly used lentiviral lnc-Hser (LV-lnc-Hser) for over-expression in primary HCs and AML12 cells. Recombinant TGFβ was then used to treat the cells after infection with LV-lnc-Hser or LV-Control, and total RNA was extracted for detecting the expression of apoptotic and EMT-related genes. The results showed that the expression of the pro-fibrotic genes α-SMA, Col1α1 and MMP2, the pro-apoptosis genes BAX and BAD, and the mesenchymal marker genes N-Cadherin, Vimentin, Fibronectin, Twist and Snail1 was markedly increased, whereas the expression of the anti-apoptosis gene Bcl2, the pro-proliferation genes Ki67 and Pcna, and the epithelial marker gene E-cadherin was significantly decreased upon TGFβ treatment. However, forced lnc-Hser expression abrogated TGFβ-induced dysregulation of these genes in primary HCs and AML12 cells assessed by qRT-PCR and western blot (Figure 3A-C and Figure S3A-D). Flow cytometry (FACS) analysis further confirmed that TGFβ stimulation dramatically induced AML12 apoptosis, however, forced lnc-Hser expression markedly suppressed TGFβ-induced apoptosis (Figure S3E). On the other hand, knockdown of lnc-Hser obviously up-regulated the expression of the pro-fibrotic genes, the pro-apoptosis genes, and the mesenchymal marker genes, while down-regulated the expression of the anti-apoptosis gene, pro-proliferation genes, and the epithelial marker gene in primary HCs and AML12 cells (Figure 3D-F and Figure S4A-E). Moreover, we generated stable knockdown of lnc-Hser using the CRISPR/Cas9 system with guide RNA pairs targeted to the promoter region and intron 1 of lnc-Hser in AML12 cells. As expected, the above results could be confirmed in lnc-Hser-silenced AML12 cells (Figure S5A-E). Additionally, the expression of the pro-apoptosis genes and the mesenchymal marker genes in the primary HCs isolated from lnc-Hser-shRNA-CCl_4_ mice exhibited a profound enhancement, but the expression of the anti-apoptosis gene and the pro-proliferation genes demonstrated a remarkable reduction, in comparison with the NC-CCl_4_ mice (Figure S6A). Taken together, these results suggest that silencing lnc-Hser induces the apoptosis and EMT of HCs.

### lnc-Hser is indirectly involved in the activation of HSCs

**Figure 4:**
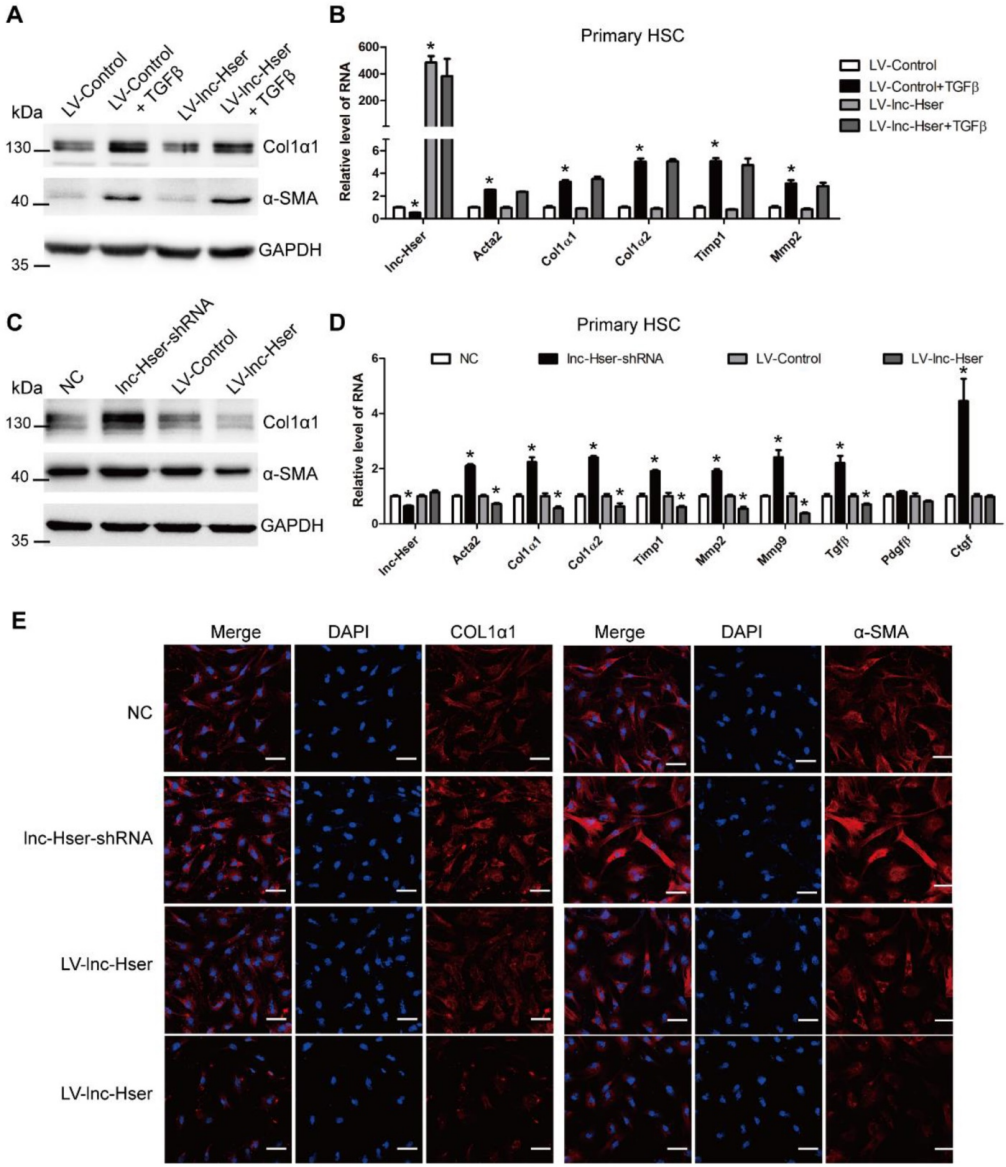
** lnc-Hser is indirectly involved in the activation of HSCs.** (A, B) Primary HSCs were infected with LV-lnc-Hser for 72 h and further treated with 10 ng/ml TGFβ for additional 24 h. The protein level of α-SMA and Col1α1 was detected by western blot. GAPDH was used as an internal control (A). The RNA level of *lnc-Hser, Acta2, Col1α1, Col1α2, Mmp2* and *Timp1* was detected by qRT-PCR (B). (C-E) The conditioned medium from control, lnc-Hser -silenced or -over-expressed AML12 cells was used to treat primary HSC at day 2. After 48 hours incubation, the protein level of α-SMA and Col1α1 was detected by western blot. GAPDH was used as an internal control (C). The RNA level of *lnc-Hser, Acta2, Col1α1, Col1α2, Timp1, Mmp2, Mmp9, Tgfβ, Pdgfβ* and *Ctgf* was detected by qRT-PCR (D). The expression of α-SMA and Col1α1 was determined by confocal microscopy. DAPI stained nuclei blue; scale bar = 50 μm. The data are expressed as the mean ± SD for at least triplicate experiments, * p<0.05.

Activation of HSCs is the most principal cellular player promoting synthesis and deposition of ECM proteins in response to accumulated levels of inflammatory signals derived from damaged parenchymal cells 5. The *in vivo* data showed that knockdown of lnc-Hser aggravated CCl_4_-induced liver fibrosis. Therefore, we investigated the effect of lnc-Hser on the activation of HSCs. Since the expression of lnc-Hser in primary HSCs is quite low, we over-expressed lnc-Hser using LV-lnc-Hser in the cells and subsequently treated them with TGFβ. The results showed that the expression of the predominant fibrotic genes including α-SMA, Col1α1, Col1α2, TIMP1 and MMP2 was up-regulated upon TGFβ treatment. However, over-expression of lnc-Hser neither directly suppressed nor abrogated TGFβ-induced up-regulation of these fibrotic genes in primary HSCs assessed by western blot and qRT-PCR (Figure 4A, B), suggesting that lnc-Hser is not directly involved in the regulation of the activation of HSCs. We therefore hypothesized that down-regulated lnc-Hser in HCs during liver fibrogenesis results in the apoptosis of HCs which subsequently acts on HSCs to promote the expression of pro-fibrotic genes.

Hence, we used conditioned medium (CM) from control, lnc-Hser-silenced or lnc-Hser-over-expressed AML12 cells to treat primary HSCs. Compared with the control cells, treatment with the CM from lnc-Hser-silenced AML12 cells significantly promoted the expression of the fibrotic genes including α-SMA and Col1α1, but the administration with CM from lnc-Hser-over-expressed AML12 cells repressed the expression of these genes assessed by western blot, qRT-PCR and confocal microscopy (Figure 4C-E). Moreover, the level of TGFβ in supernatant from the cultured lnc-Hser-silenced AML12 cells and primary HCs was markedly increased (Figure S6B, C), suggesting that TGFβ is one of the factors derived from injured HCs that promotes HSCs activation. Taken together, these results clearly suggest that silencing lnc-Hser promotes HSCs activation through the signals derived from damaged HCs.

### lnc-Hser inhibits HCs apoptosis via the C5AR1-Hippo-YAP pathway

**Figure 5:**
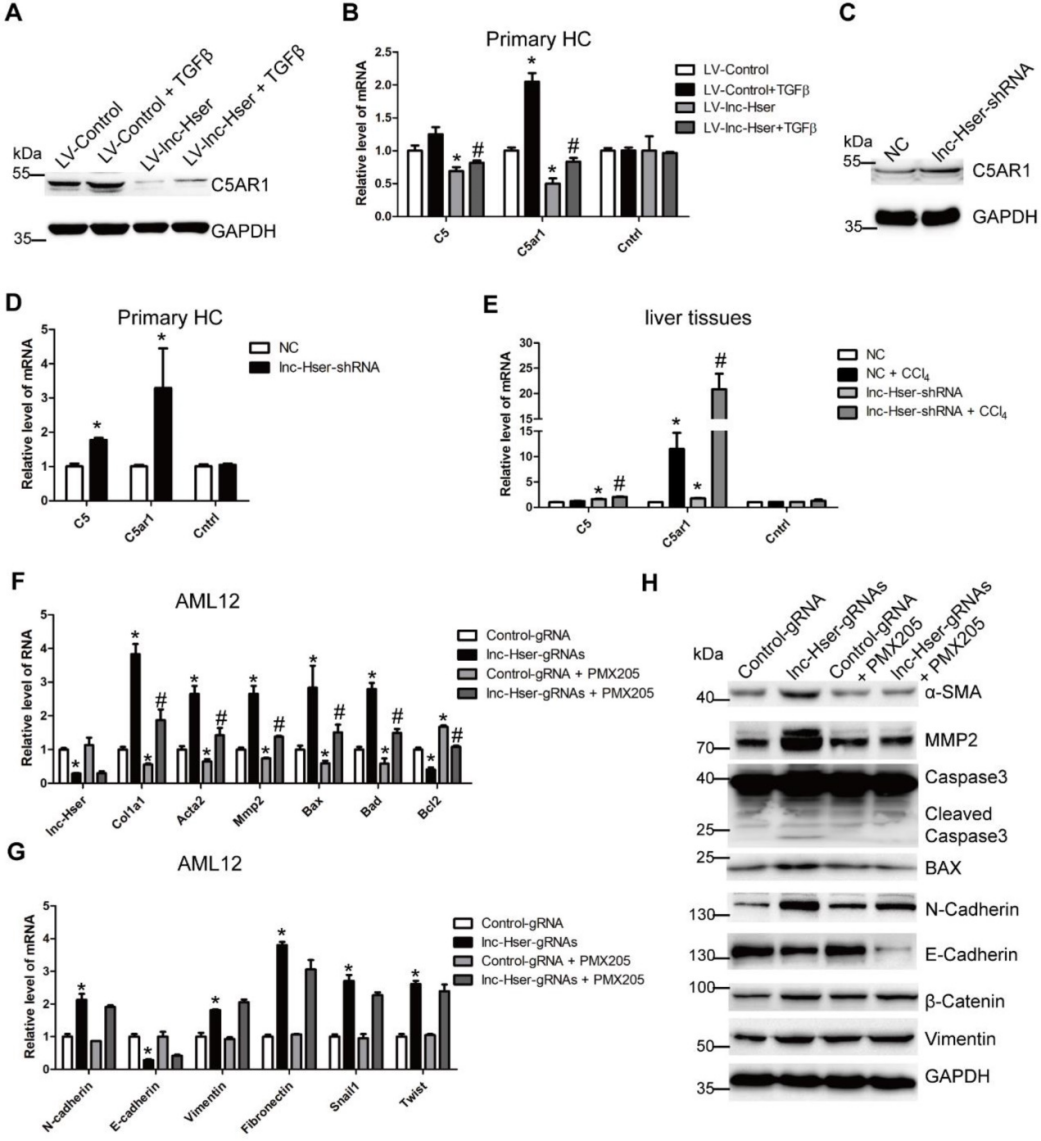
** lnc-Hser inhibits HCs apoptosis via C5AR1.** (A, B) Primary HCs were infected with LV-lnc-Hser for 72 h and further treated with 10 ng/ml TGFβ for additional 24 h. The protein level of C5AR1 was detected by western blot. GAPDH was used as an internal control (A). The mRNA level of *C5, C5ar1* and *Cntrl* was detected by qRT-PCR (B). (C) The protein level of C5AR1 in primary HCs infected with lenti-lnc-Hser-shRNA or lenti-NC was detected by western blot. GAPDH was used as an internal control. (D) The mRNA level of *C5, C5ar1* and *Cntrl* was detected in primary HCs infected with lenti-lnc-Hser-shRNA or lenti-NC by qRT-PCR. (E) Mice were treated with oil in combination with injection of lenti-NC (Negative Control, n = 10), or CCl_4_ in combination with injection of lenti-NC (NC + CCl_4_, n = 10), or oil in combination with injection of lenti-lnc-Hser-shRNA (lnc-Hser-shRNA, n = 10), or CCl_4_ in combination with injection of lenti-lnc-Hser-shRNA (lnc-Hser-shRNA + CCl_4_, n = 10). qRT-PCR analysis of *C5, C5ar1* and *Cntrl* in livers of mice in each group. (F-H) lnc-Hser was stably knocked down by the CRISPR/Cas9 system with guide RNA pairs in AML12 cells. PMX205, a specific inhibitor of C5AR1, was used to treat lnc-Hser-silenced AML12 cells for 24 h. The expression of lnc-Hser, pro-fibrogenic, apoptosis-related and EMT-related genes was detected by qRT-PCR (F, G) and western blot. GAPDH was used as an internal control (H). The data are expressed as the mean ± SD for at least triplicate experiments. *p<0.05 stands for vs LV-Control, NC or Control gRNA. #*p*<0.05 stands for vs LV-Control + TGFβ, NC+ CCl_4_ or lnc-Hser-gRNAs.

It is well known that lncRNAs work *in cis* when their effects are restricted to the chromosome from which they are transcribed, and work* in trans* when they affect genes on other chromosomes 29. To investigate the mechanism underlying lnc-Hser expression, we firstly examined whether lnc-Hser regulates its nearby genes *in cis*. The expression of Cntrl and C5, both of which are lnc-Hser neighboring genes, and C5AR1 which is a member of GPCRs family and plays an essential role in the early events leading to HCs proliferation and liver fibrosis 30, 31, was determined in either lnc-Hser over-expressed or silenced primary HCs and AML12 cells. We noticed that forced lnc-Hser expression down-regulated the expression of C5 and C5AR1 rather than Cntrl, whereas knockdown of lnc-Hser promoted the expression of both factors assessed by western blot and qRT-PCR (Figure 5A-D and Figure S7A-F). Similar results were observed in livers of the* in vivo* models (Figure 5E and Figure S7G). Since the GPCRs have been reported to function upstream of the Hippo pathway that modulates cell proliferation, differentiation and death, we then sought to explore whether lnc-Hser regulates the activation of the Hippo pathway. As shown in Figures S8 and S9, although over-expression of lnc-Hser slightly decreased the TGFβ-induced up-regulation of the compounds of the Hippo pathway including *Lats1, Yap* and *Taz* in primary HCs, *Mst1* and *Lats1* in AML12 cells, lnc-Hser had no effect on the mRNA level of these compounds. However, the phosphorylation of MST, LATS and YAP was significantly decreased in lnc-Hser-over-expressed cells, while markedly increased in lnc-Hser-silenced cells, suggesting that lnc-Hser inactivates the canonical Hippo pathway. Consistently, these results were also confirmed in the livers and primary HCs isolated from the *in vivo* model mice (Figure S10A-C). We then asked whether lnc-Hser ameliorates the apoptosis or EMT of HCs via C5AR1. PMX205, the specific inhibitor of C5AR1, was applied to treat the lnc-Hser-silenced AML12 cells and the expression of the pro-fibrotic, apoptosis and EMT associated genes was subsequently detected by western blot and qRT-PCR. Strikingly, PMX205 abrogated lnc-Hser silencing -induced up-regulation of both the pro-fibrotic and the pro-apoptotic genes but not the mesenchymal marker genes, suggesting that the inhibition of C5AR1 by lnc-Hser could suppress the apoptosis of HCs (Figure 5F-G). Moreover, FACS analysis revealed that the apoptosis induced by lnc-Hser silencing was abrogated by PMX205 (Figure S10D). Taken together, these results clearly suggest that lnc-Hser silencing promotes HCs apoptosis via the C5AR1-Hippo-YAP pathway.

### lnc-Hser inhibits HCs EMT via the Notch pathway

**Figure 6:**
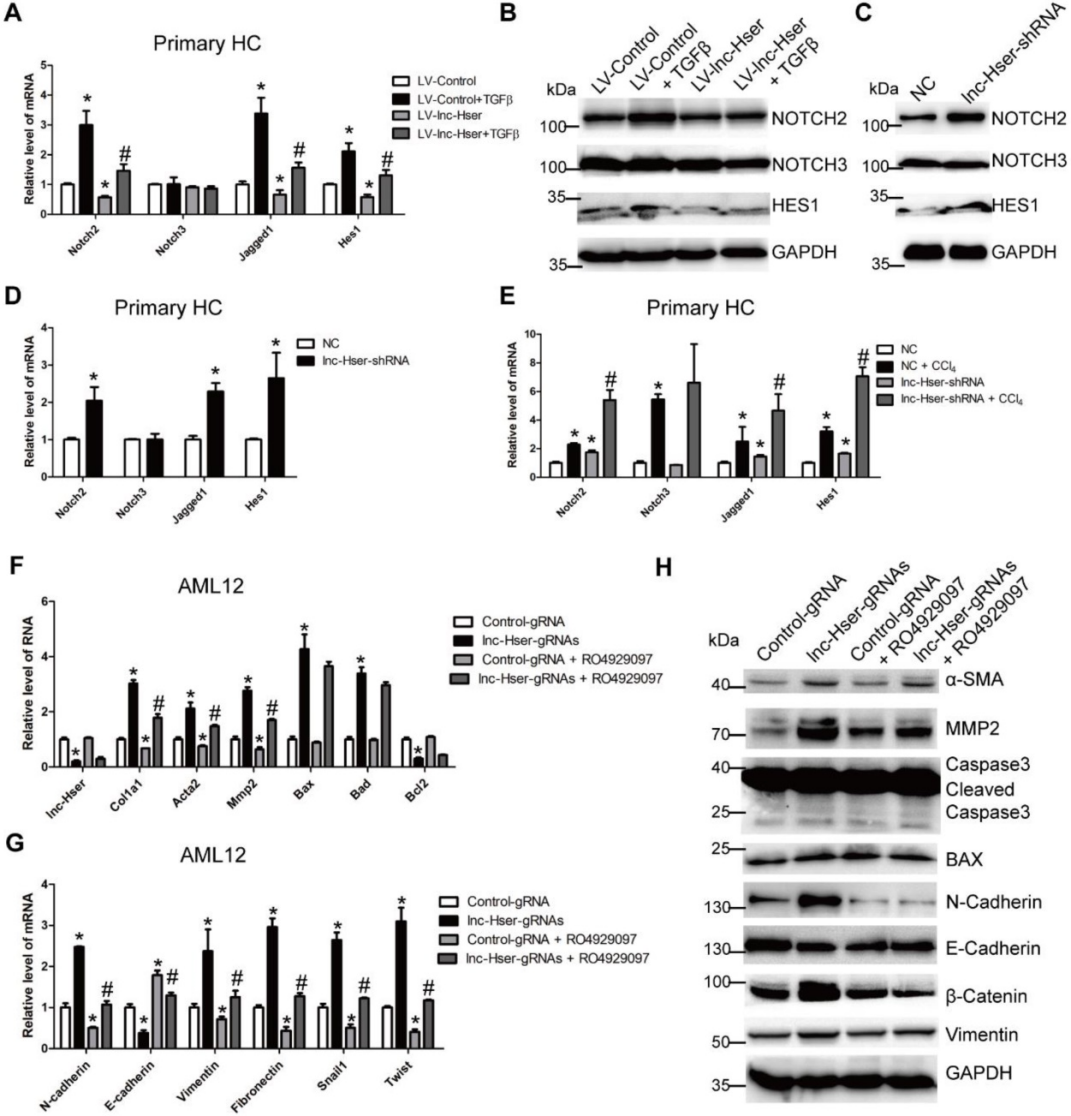
** lnc-Hser inhibits HCs EMT via the Notch pathway.** (A, B) Primary HCs were infected with LV-lnc-Hser for 72 h and further treated with 10 ng/ml TGFβ for additional 24 h. The mRNA level of *Notch2, Notch3, Jagged1* and *Hes1* was detected by qRT-PCR (A). The protein level of NOTCH2, NOTCH3 and HES1 was detected by western blot. GAPDH was used as an internal control (B). (C) The protein level of NOTCH2, NOTCH3 and HES1 in primary HCs infected with lenti-lnc-Hser-shRNA or lenti-NC was detected by western blot. GAPDH was used as an internal control. (D) The mRNA level of *Notch2, Notch3, Jagged1* and *Hes1* in primary HCs infected with lenti-lnc-Hser-shRNA or lenti-NC was detected by qRT-PCR. (E) Mice were treated with oil in combination with injection of lenti-NC (Negative Control, n = 10), or CCl_4_ in combination with injection of lenti-NC (NC + CCl_4_, n = 10), or oil in combination with injection of lenti-lnc-Hser-shRNA (lnc-Hser-shRNA, n = 10), or CCl_4_ in combination with injection of lenti-lnc-Hser-shRNA (lnc-Hser-shRNA + CCl_4_, n = 10). qRT-PCR analysis of *Notch2, Notch3, Jagged1* and *Hes1* in the primary HCs isolated from mice in each group. (F-H) lnc-Hser was stably knocked down by the CRISPR/Cas9 system with guide RNA pairs in AML12 cells. The γ-secretase inhibitor RO4929097 was used to treat lnc-Hser-silenced AML12 cells for 24 h. The expression of lnc-Hser, pro-fibrogenic, apoptosis-related and EMT-related genes was detected by qRT-PCR (F, G) and western blot. GAPDH was used as an internal control (H). The data are expressed as the mean ± SD for at least triplicate experiments. *p<0.05 for stands vs LV-Control, NC or Control gRNA. #*p*<0.05 stands for vs LV-Control + TGFβ, NC + CCl_4_ or lnc-Hser-gRNAs.

Increasing evidences suggest that Notch signaling contributes to the progression of liver fibrosis via inducing the EMT 7, 8. We therefore considered the hypothesis that lnc-Hser may inhibit the EMT of HCs through the Notch pathway. The level of Notch2, Notch3, Jagged1 and Hes1 was detected in lnc-Hser-over-expressed and lnc-Hser-silenced primary HCs and AML12 cells. The results showed that forced lnc-Hser expression inhibited the expression of Notch2, Jagged1 and Hes1 rather than Notch3, whereas lnc-Hser silencing promoted the expression of these genes (Figure 6A-D and Figure S11A-F). These results were also confirmed in the livers and primary HCs isolated from the *in vivo* model mice (Figure 6E and Figure S12A, B). To further investigate whether lnc-Hser suppresses the EMT of HCs through the Notch pathway, the signaling was blocked by a γ-secretase inhibitor RO4929097 in lnc-Hser-silenced AML12 cells. Subsequently, the expression of the genes related to fibrosis, apoptosis and EMT was detected by qRT-PCR and western blot. Interestingly, blocking the Notch pathway abrogated lnc-Hser silencing -induced up-regulation of both the pro-fibrotic and the mesenchymal marker genes but not the pro-apoptosis genes (Figure 6F-G). Taken together, our data demonstrate that lnc-Hser inhibits HCs EMT via the Notch pathway.

### Potential role of lnc-HSER in human liver fibrosis

**Figure 7:**
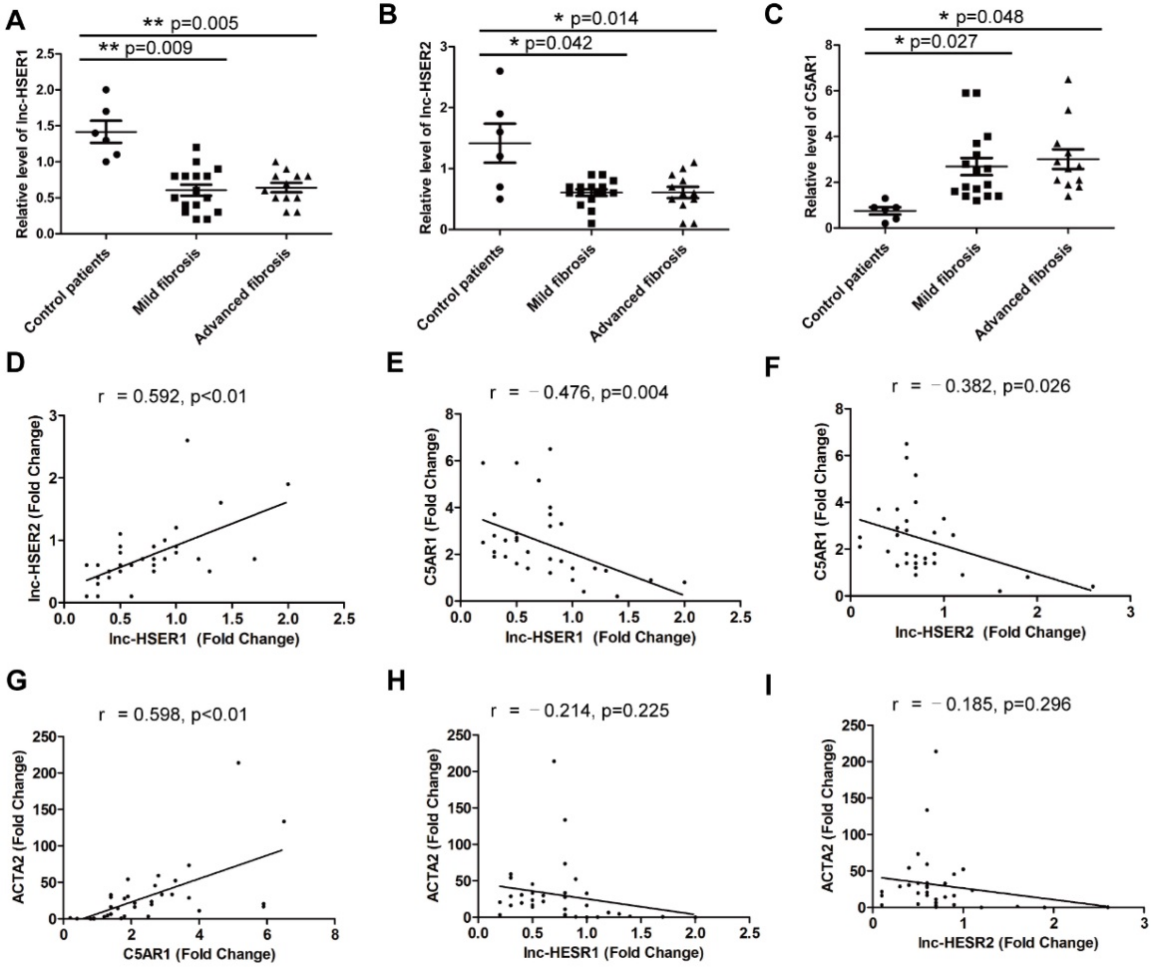
** Potential role of lnc-HSER in human liver fibrosis.** (A-C) qRT-PCR analysis of *lnc-HSER1, lnc-HSER2* and *C5AR1* in liver samples from healthy (n = 6), mild fibrosis (n = 16) and advanced fibrosis (n = 12) patients. (D-I) The correlations of lnc-HSER1, lnc-HSER2, C5AR1 and ACTA2 were assessed using Pearson correlation analysis, n = 34. The data are expressed as the mean ± SD, *p<0.05.

Although the function of lncRNA is important, the rapid sequence evolution of lncRNAs presents a challenge to identify functional counterparts between species. To address the question if our results with mice can be transferable to the human situation, the human ortholog lnc-HSER is needed to be identified. However, sequence analysis indicated that the full length of lnc-Hser was poorly conserved across species. Taking advantage of the presence of the conserved neighboring genes Cntrl and C5, we found that ENST00000466280, ENST00000460578, ENST00000489802, ENST00000480188 and NR_148450 were most likely to be the human homolog of lnc-Hser, all of which are located on human chromosome 9. Since the level of lnc-Hser was quite high in normal livers and was down-regulated in fibrotic livers, we next measured the expression of these five human lncRNAs in 6 healthy livers, 16 mild fibrotic livers (F1-F2) and 12 advanced fibrotic livers (F3-F4). The results showed that the transcript of ENST00000466280, ENST00000460578 and ENST00000480188 was enriched in healthy livers, but the level of ENST00000460578 and ENST00000480188 was significantly reduced in fibrotic livers (Figure 7A, B). Thus, we referred to ENST00000460578 as lnc-HSER1 and ENST00000480188 as lnc-HSER2. Moreover, the expression of C5AR1 was markedly increased in fibrotic livers, but not further increased with the progression of fibrosis (Figure 7C). In addition, lnc-HSER1 was found to be positively correlated with lnc-HSER2 (Figure 7D). A correlation of C5AR1 with lnc-HSER1, lnc-HSER2 and ACTA2 was also observed (Figure 7E-G). However, neither lnc-HSER1 nor lnc-HSER2 was correlated with ACTA2 (Figure 7H, I). To further investigate the role of lnc-HSER1 and lnc-HSER2, we over-expressed both lncRNAs in L02 cells and subsequently treated the cells with TNF-α. The results showed that the expression of the pro-inflammation genes IL-6, IL-1β and TNF-α, and the pro-apoptosis genes BAX and BAD was significantly up-regulated, whereas that of the mesenchymal marker genes N-Cadherin, Vimentin, Fibronectin, Twist and Snail1 was slightly up-regulated upon TNF-α treatment. Strikingly, forced lnc-HSER1 or lnc-HSER2 expression abrogated TNF-α-induced dysregulation of these genes (Figure S13A-D). Taken together, these data support the notion that lnc-HSER1/2 are significantly enriched in normal human livers but decreased in fibrotic livers and display a potential anti-fibrosis role.

## Discussion

Liver fibrosis is a progressive pathologic process that involves deposition of ECM leading to distorted architecture and culminating in cirrhosis, which is one of the major health burdens worldwide 2. However, the molecular basis of liver fibrosis is incompletely understood, which has limited the identification of therapeutic targets. In the current study, we identified a HCs-specifically expressed lnc-Hser that was decreased in human and mice fibrotic livers as well as primary HCs of mice developing liver fibrosis. We showed that knockdown of lnc-Hser induced the apoptosis and EMT of HCs* in vitro*, and aggravated CCl_4_-induced liver fibrosis *in vivo*. Although the* in vivo* data showed that simply knockdown of lnc-Hser did not induce liver fibrosis, the expression of genes related to liver fibrosis including Col4α5, Timp1 and Mmp2, the hepatic inflammation including Tnf-α, Mcp1 and Il-6, the mesenchymal markers N-Cadherin and β-Catenin was increased in livers of lnc-Hser-shRNA mice versus NC mice. The reason for lnc-Hser silencing did not induce significant liver fibrosis may be due to the fact that liver fibrogenesis is a complex process involved in many key factors, and the time period for hepatic fibrogenesis of the mice was only four weeks during which the liver had significant regeneration and self-renewal capacity to counteract the injury caused by silencing lnc-Hser. In addition, knockdown of lnc-Hser promoted HSCs activation through the signals derived from injured HCs. Mechanistically, we demonstrated that lnc-Hser inhibited HCs apoptosis via the C5AR1-Hippo-YAP pathway and suppressed HCs EMT via the Notch pathway. All these data support our conclusion that lnc-HSER is a novel biomarker for damaged HCs and a potential target for anti-fibrotic therapy.

It is well accepted that the apoptosis of HCs is a critical initiator of fibrosis by triggering HSCs activation either directly by the phagocytosis of the apoptotic bodies which promote the secretion of pro-inflammatory and pro-fibrogenic cytokines, or indirectly by the generation of damage-associated molecular patterns 2, 5. However, the occurrence of HCs EMT in liver fibrosis remains controversial, for instance, Zeisberg et al. demonstrated that HCs undergo EMT in response to CCl_4_ *in vivo* 32, while Taura and colleagues employed a similar transgenic model and reported that HCs did not undergo EMT in CCl_4_-induced liver fibrosis 33. Strikingly, several groups demonstrated that primary HCs and AML12 cells, when treated with the classical EMT inducer TGF-β, undergo EMT genotypic and phenotypic changes* in vitro* 8, 34, 35. Consistently, our data showed that lnc-Hser silencing induced the apoptosis and EMT of HCs* in vitro*. On the other hand, the expression of the pro-apoptosis genes and the mesenchymal marker genes in the livers and primary HCs isolated from lnc-Hser-shRNA-CCl_4_ mice exhibited a profound increase, while the epithelial marker gene E-cadherin was not decreased. The reason for this could be when EMT initiates the epithelial cells to gain some phenotypes of the mesenchymal cells, for instance, up-regulated expression of N-cadherin and ECM, but still keeps some phenotypes of the epithelium. This is the so-called partial EMT. In this study, when EMT initiates during hepatic fibrogenesis, a reversible wound healing process, the HCs have to keep the cellular junctions to maintain the architecture of the hepatic lobule by normal expression of E-cadherin. However, the HCs have gained some mesenchymal phenotypes including over-expressed N-cadherin, β-catenin and Vimentin. Interestingly, our *in vivo* and* in vitro* results further revealed that the apoptosis and EMT of HCs were not co-localized, suggesting when lnc-Hser was silenced there are two populations of the cells among which one undergoes apoptosis and the other survived one undergoes EMT simultaneously. Hence, the inhibition of HCs apoptosis and EMT may be a therapeutic approach for the resolution of liver fibrosis.

Accumulating progresses have been made in better understanding the underlying mechanisms of liver fibrosis with recent major investigations focused on the pathways including TGFβ1, Notch and Hippo, involved in initiating HCs apoptosis and EMT in the pathogenesis of liver fibrosis. Notably, it has been reported that Notch signaling contributes to the progression of liver fibrosis via inducing the EMT 7, 8, and the EMT of *in vitro* cultured primary HCs could be blocked in the presence of several inhibitors including a Notch inhibitor 9. Consistently, our results revealed that the up-regulated expression of the pro-fibrotic and the mesenchymal marker genes but not the pro-apoptosis genes induced by lnc-Hser silencing was abrogated by the γ-secretase inhibitor, suggesting that lnc-Hser inhibits HCs EMT via the Notch pathway. However, the role of YAP/TAZ in HCs apoptosis and proliferation remains controversial. We and others have demonstrated that the expression of YAP/TAZ was increased in fibrotic tissues and activated HSCs 12, 13, suggesting that YAP/TAZ activation is a critical driver of HSCs activation and subsequently induces liver fibrosis. Additionally, Lee et al. showed that YAP activation induced by inactivation of the Hippo pathway in HCs induced massive p53-dependent cell senescence/death 14. Wang et al. demonstrated that silencing of HCs TAZ in murine models of nonalcoholic steatohepatitis (NASH) prevented or reversed hepatic inflammation, HCs death, and fibrosis 36. However, it also has been reported that knockout of YAP in HCs resulted in increased HCs apoptosis and subsequently induced liver fibrosis *in vivo* and* in vitro* 15. Similar results were also reported by Bai et al 16. In addition, liver regeneration requires activated YAP and regenerative growth is blocked by preventing YAP activation, indicating that YAP promotes HCs proliferation 37. In our study, we demonstrated that the canonical Hippo pathway was activated in damaged HCs and resulted in YAP inactivation.

LncRNAs represent a group of transcripts longer than 200 nucleotides that are 5' capped and polyadenylated like most mRNAs, yet this class of transcripts has limited coding potential. Emerging evidence has demonstrated that lncRNAs were expressed in a cell- or tissue-specific manner and involved in a variety of cellular processes including differentiation, proliferation, and apoptosis 22, 38-40. In this study, we identified a novel lncRNA that was expressed with a highest level in livers and HCs but not in HSCs, LSECs and KCs, and was therefore named as lnc-Hser. However, the results of RACE showed that lnc-Hser is a 588-nucleotide transcript with three exons inconsistent with the sequence in the ensemble database. Both the transcripts share the same transcription start site and the first two exons, whereas the length of the third exon of lnc-Hser is longer than that of ENSMUST00000154817 displayed in database. The discrepancy may be due to the existence of different isoforms in various tissues. Although sequence analysis revealed no clear orthologous lnc-Hser in the human genome which is consistent with the idea that lncRNAs are less conserved than protein-coding genes, we found, by taking advantage of the presence of the conserved nearby genes Cntrl and C5, that five lncRNAs located on human chromosome 9 were most likely to be the human homologs of lnc-Hser. Additionally, we determined to regard the lncRNAs ENST00000460578 and ENST00000480188 as lnc-HSERs through detecting the transcripts of these lncRNAs in human livers and subsequently investigating the role of them in cells. Thus, it might be a way to discover homologous transcripts across species by the adjacent conserved genes, the expression profiles and the similar functions.

It is well known that lncRNAs work *in cis* when their effects are restricted to the chromosome from which they are transcribed, and work *in trans* when they affect genes on other chromosomes 29, 40. In the current study, our data showed that lnc-Hser down-regulated the level of C5 rather than Cntrl* in cis*. Moreover, lnc-Hser could also inhibit the expression of Notch2, Jagged1, Hes1 and C5AR1 which is a GPCR family member and functions upstream of the Hippo pathway 11, but not Notch3, *in trans*. Since lnc-Hser was mainly located in the nucleus of HCs, we also performed RIP assay in AML12 cells and livers to pull down endogenous RNAs associated with SUZ12, an important subunit of PRC2, which physically associates with twenty percent of all human lncRNAs 37. Our data also showed that a significant enrichment of lnc-Hser rather than β-actin with the SUZ12 (Figure S14A, B). Moreover, ChIP analysis was performed and the data demonstrated that over-expression of lnc-Hser increased the binding of SUZ12 across the promoters of C5ar1, Notch2 and Hes1 (Figure S14C), suggesting that lnc-Hser binds PRC2 and subsequently represses the transcription of the target genes. Additionally, ChIP results showed that TGFβ promoted the binding of SUZ12 across the lnc-Hser promoters (Figure S14D).

In conclusion, our data identified lnc-HSER as a novel regulator of C5AR1-Hippo-YAP and Notch pathways in liver fibrosis, suggesting that lnc-HSER might be a novel biomarker and a candidate of anti-fibrotic targets.

## Supplementary Material

Supplementary figures and tables.

## Abbreviations

lncRNAs: long non-coding RNAs
HC: hepatocyte
HSC: hepatic stellate cell
ECM: extracellular matrix
EMT: epithelial-mesenchymal transition
NICD: Notch intracellular domain
GPCRs: G-protein-coupled receptors
LSEC: liver sinusoidal endothelial cell
KC: Kupffer cell
CCl_4_: carbon tetrachloride
BDL: bile duct ligation
α-SMA: alpha-smooth muscle actin
FACS: flow cytometry
CM: conditioned medium
TGFβ: transforming growth factor-β
