# Supplementary material for: The hepatocyte-specifically expressed lnc-HSER alleviates hepatic fibrosis by inhibiting hepatocyte apoptosis and epithelial-mesenchymal transition
Source: Theranostics. 2019 Oct 12;9(25):7566–82. doi: 10.7150/thno.36942 (PMC6831459; doi:10.7150/thno.36942)
Supplement: Supplementary file 1 — Supplementary figures and tables. [file thnov09p7566s1.pdf]

The hepatocyte-specifically expressed lnc-HSER alleviates hepatic fibrosis by inhibiting hepatocyte apoptosis and epithelial-mesenchymal transition

Kun Zhang[1, #], Mengxia Zhang[1, #], Qingbin Yao[1, #], Xiaohui Han[1, #], Yanmian Zhao[1], Lina Zheng[1], Guantong Li[2], Qi Liu[1], Yanan Chang[3], Peijun Zhang[4], Hongmei Cui[1], Zhemin Shi[1], Ting Chen[1], Zhi Yao[5], Tao Han[2], Wei Hong[1, *]

[1]Department of Histology and Embryology, Key Laboratory of Immune Microenvironment and Disease of Ministry of Education, Tianjin Key Laboratory of Cellular and Molecular Immunology, School of Basic Medical Sciences, Tianjin Medical University, Tianjin, China

[2]The Third Central Clinical College of Tianjin Medical University, Department of Hepatology and Gastroenterology, Tianjin Third Central Hospital, Tianjin Key Laboratory of Artificial Cells, Artificial Cell Engineering Technology Research Center of Public Health Ministry, Tianjin, China

[3]Department of Pathology, Institute of Hematology and Blood Diseases Hospital, Chinese Academy of Medical Sciences and Peking Union Medical College, Tianjin, China

[4]Tianjin Central Hospital of Gynecology Obstetrics, Tianjin, China

[5]Department of Immunology, Key Laboratory of Immune Microenvironment and Disease of Ministry of Education, Tianjin Key Laboratory of Cellular and Molecular Immunology, School of Basic Medical Sciences, Tianjin Medical University, Tianjin, China

[#]These authors contributed equally to this work.

[*]**Corresponding authors:** Wei Hong, Department of Histology and Embryology, School of Basic Medical Sciences, Tianjin Medical University, Tianjin, China. E-mail: hongwei@tmu.edu.cn.

Supplementary Information

Supplementary Figures

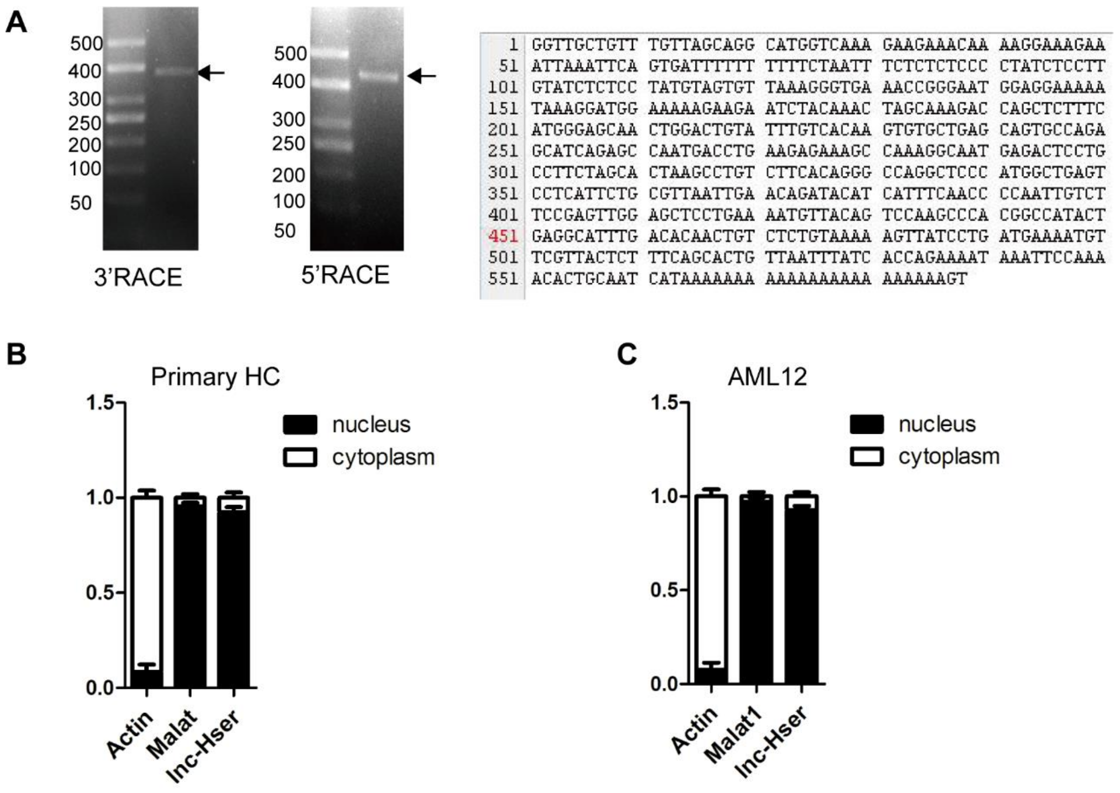

Fig. S1, related to Fig. 1. (A) Agarose gel electrophoresis of PCR products from the 5'-RACE procedure and 3'-RACE procedure. The molecular weight markers (base pairs) are indicated on the side. The major PCR product is marked with an arrow. Nucleotide sequence of the full-length lnc-Hser was confirmed by RACE in mouse livers. (B, C) RNA was extracted from the nuclei or cytoplasm of primary HCs and AML12 cells. 1 µg of RNA was used for the qRT-PCR analysis of *lnc-Hser, Malat1* (nuclear retained), and *β-actin* mRNAs (cytoplasm retained). The data are expressed as the mean ± SD for at least triplicate experiments.

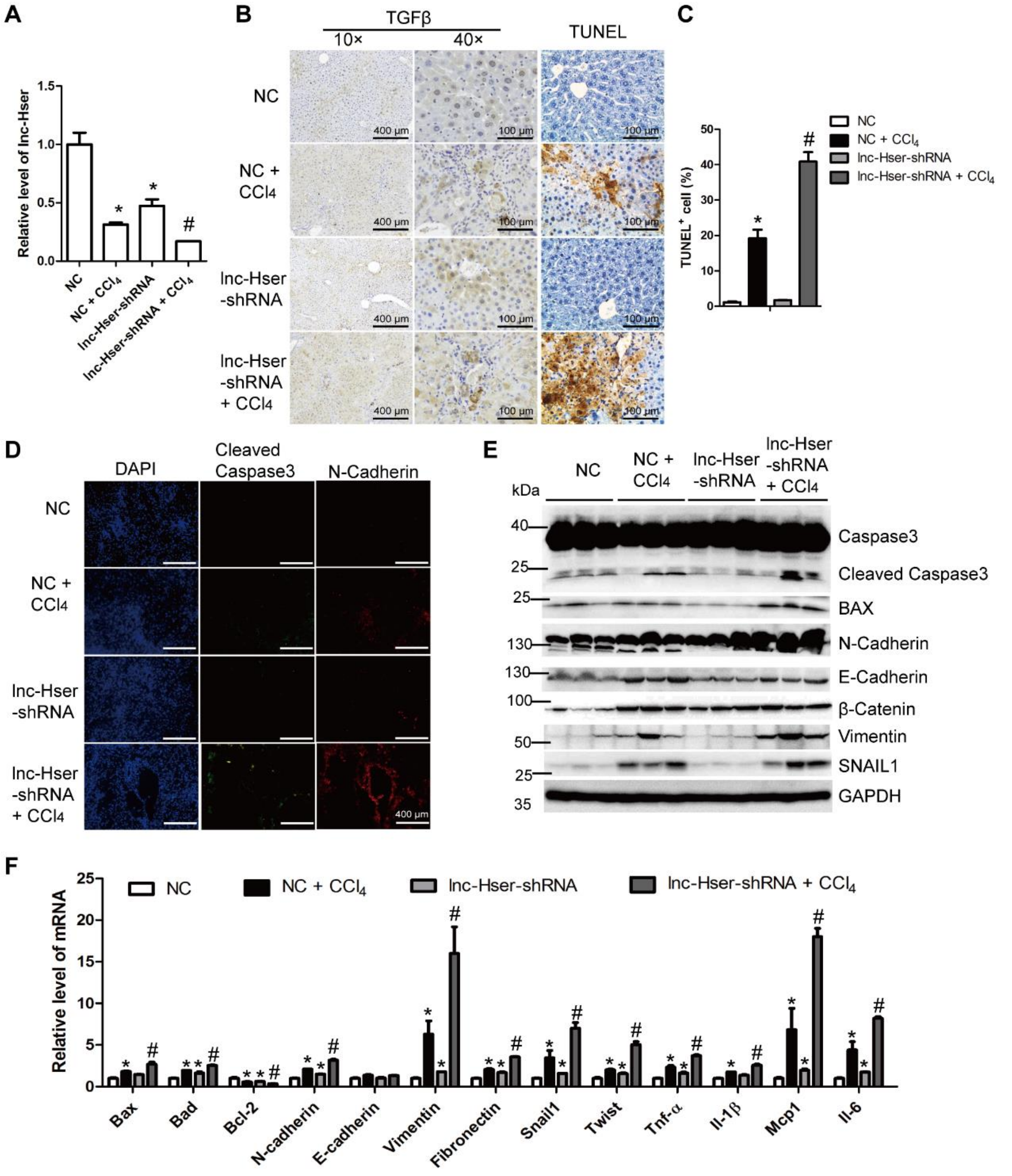

Fig. S2, related to Fig. 2. Mice were treated with oil in combination with injection of lenti-NC (Negative Control, n = 10), or CCl_4 in combination with injection of lenti-NC (NC + CCl_4, n = 10), or oil in combination with injection of lenti-lnc-Hser-shRNA (lnc-Hser-shRNA, n = 10), or CCl_4 in combination with injection of lenti-lnc-Hser-shRNA (lnc-Hser-shRNA + CCl_4, n = 10). (A) The expression of *lnc-Hser* in livers of each group was examined by qRT-PCR. (B) Liver fibrosis was evaluated by IHC for TGFβ and TUNEL staining; scale bar = 400 μm for 10× and 100 μm for 40×. (C) Quantification of TUNEL staining. (D) The expression and location of cleaved Caspase3 and N-Cadherin were determined by IHC (Frozen); scale bar = 400 μm; (E) The

protein level of total and cleaved Caspase3, BAX, N-Cadherin, E-Cadherin, β-Catenin, Vimentin and SNAIL1 was determined by western blot. GAPDH was used as an internal control. (F) The mRNA level of the apoptosis-related, pro-inflammation and EMT-related genes was determined by qRT-PCR. The data are expressed as the mean ± SD for at least triplicate experiments. *$p<0.05$ stands for vs NC. #$p<0.05$ stands for vs NC + CCl_4.

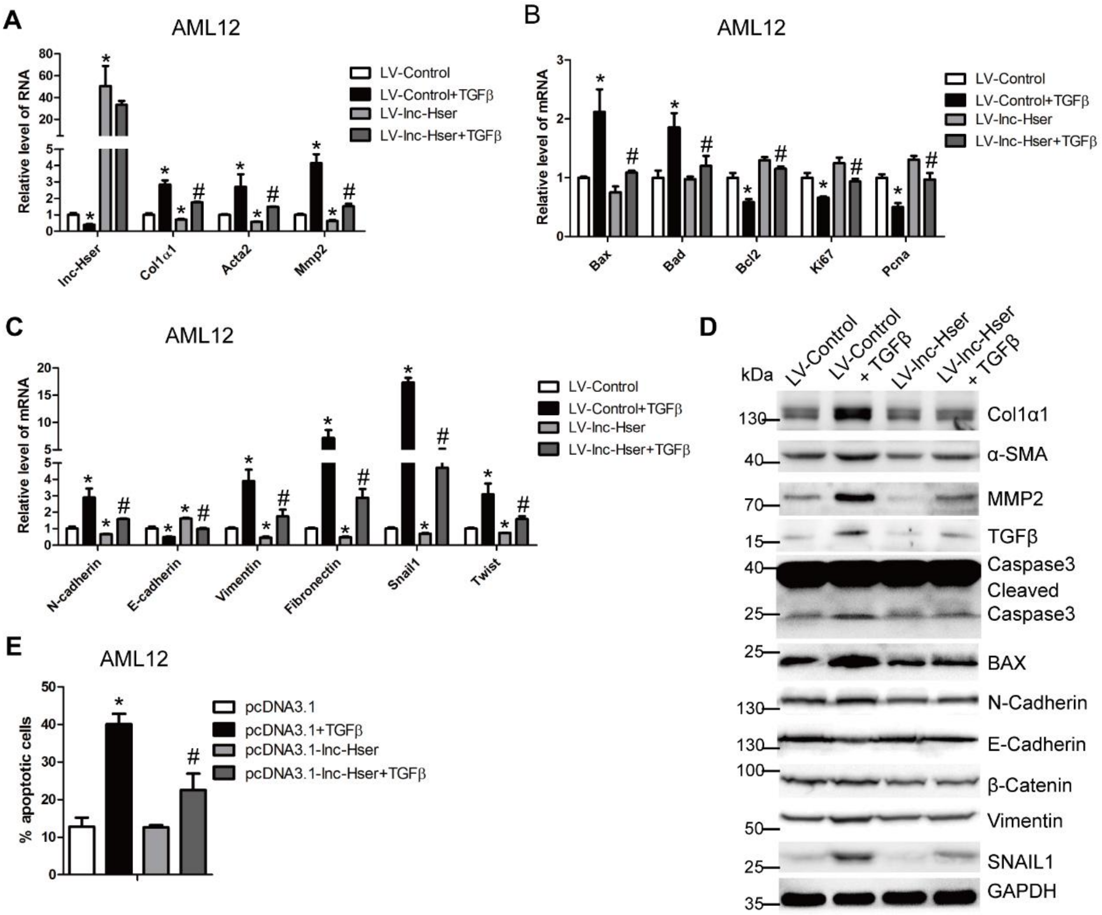

Fig. S3, related to Fig. 3. (A-D) AML12 cells were infected with LV-lnc-Hser for 72 h and further treated with 10 ng/ml TGFβ for additional 24 h. The RNA level of *lnc-Hser,* pro-fibrogenic genes (A), apoptosis and proliferation -related genes (B) and EMT-related genes (C) was detected by qRT-PCR. The protein level of α-SMA, Col1α1, MMP2, total and cleaved Caspase3, BAX, N-Cadherin, E-cadherin, Vimentin, β-Catenin and SNAIL1 was detected by western blot. GAPDH was used as an internal control (D). (E) AML12 cells were transfected with pcDNA3.1-lnc-Hser or pcDNA3.1 for 48 h and further treated with 10 ng/ml TGFβ for additional 24 h. Cell apoptosis was determined by FACS analysis. The data are expressed as the mean ± SD for at least triplicate experiments. *p<0.05 stands for vs LV-Control or pcDNA3.1. #p<0.05 stands for vs LV-Control + TGFβ or pcDNA3.1 + TGFβ.

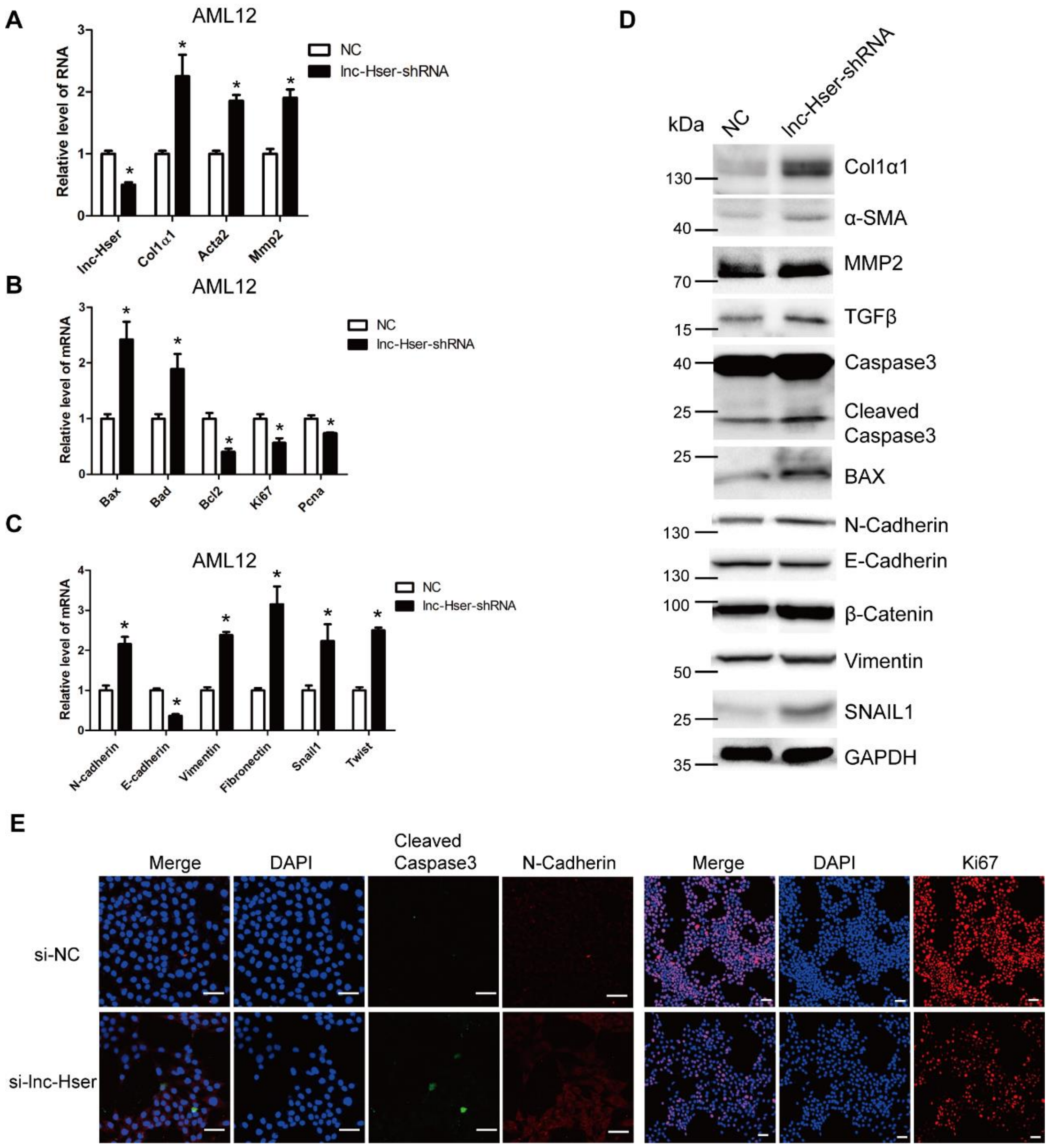

Fig. S4, related to Fig. 3. (A-D) The expression of lnc-Hser, pro-fibrogenic genes (A), apoptosis and proliferation -related genes (B) and EMT-related genes (C) was detected in AML12 cells infected with lenti-lnc-Hser-shRNA or lenti-NC by qRT-PCR (A-C) and western blot. GAPDH was used as an internal control (D). (E) AML12 cells were transfected with siRNA-lnc-Hser or si-NC for 36 h, the expression and location of cleaved Caspase3, N-Cadherin and Ki67 was determined by confocal microscopy. DAPI stained nuclei blue; scale bar = 50 μm. The data are expressed as the mean ± SD for at least triplicate experiments, *p<0.05.

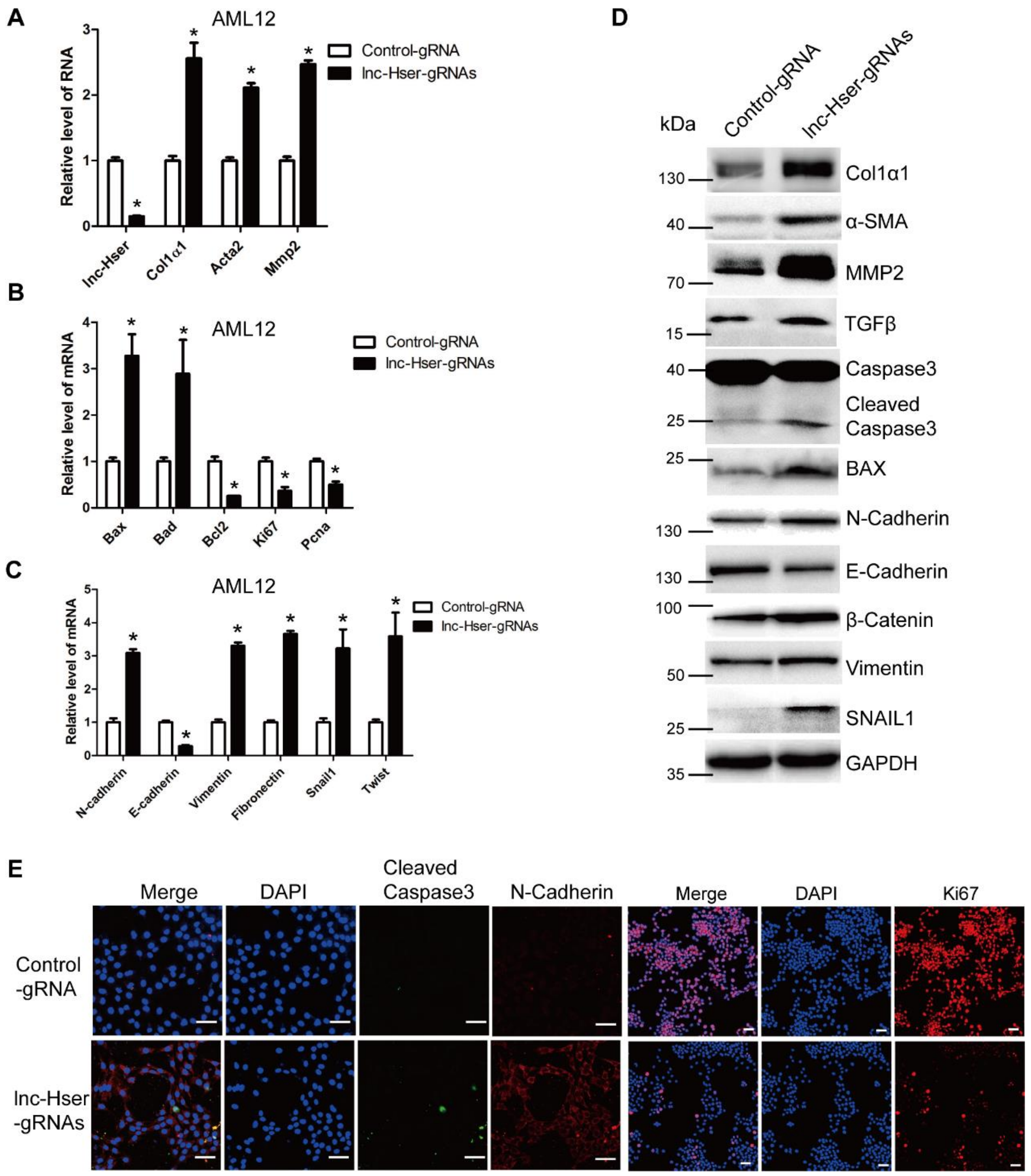

Fig. S5, related to Fig. 3. The expression of lnc-Hser was stably knocked down by the CRISPR/Cas9 system with guide RNA pairs in AML12 cells. (A-E) The expression of lnc-Hser, pro-fibrogenic genes (A), apoptosis and proliferation -related genes (B) and EMT-related genes (C) was detected in lnc-Hser-silenced AML12 cells by qRT-PCR (A-C) and western blot. GAPDH was used as an internal control (D). The expression and location of cleaved Caspase3, N-Cadherin and Ki67 was determined by confocal microscopy. DAPI stained nuclei blue; scale bar = 50 μm (E). The data are expressed as the mean ± SD for at least triplicate experiments, *p<0.05.

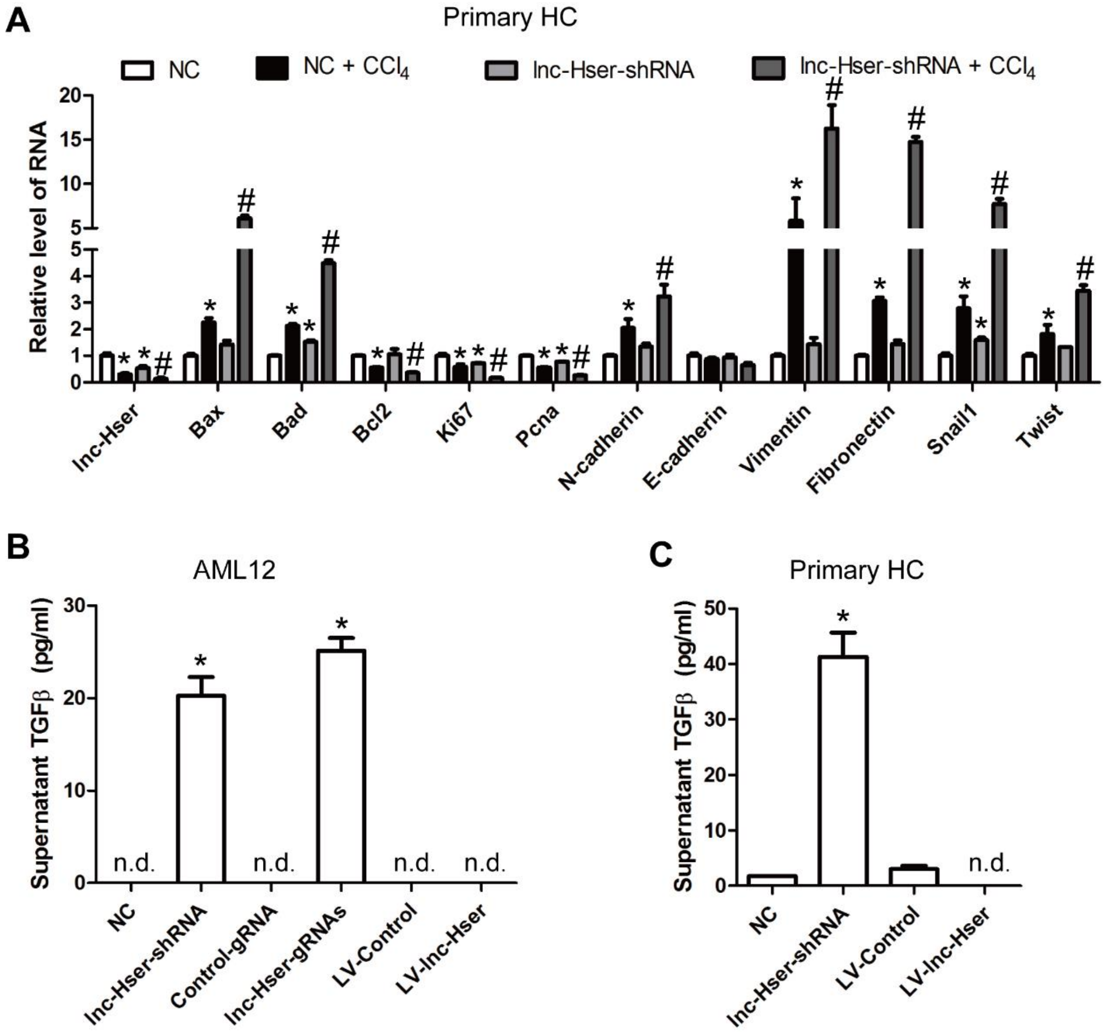

Fig. S6, related to Fig. 3, 4. (A) Mice were treated with oil in combination with injection of lenti-NC (Negative Control, n = 10), or CCl_4 in combination with injection of lenti-NC (NC + CCl_4, n = 10), or oil in combination with injection of lenti-lnc-Hser-shRNA (lnc-Hser-shRNA, n = 10), or CCl_4 in combination with injection of lenti-lnc-Hser-shRNA (lnc-Hser-shRNA + CCl_4, n = 10). qRT-PCR analysis of *lnc-Hser,* apoptosis and proliferation -related genes and EMT-related genes level in the primary HCs isolated from mice in each group. (B) The level of TGFβ in supernatant from Control, lnc-Hser-silenced and lnc-Hser-over-expressed AML12 and primary HCs was detected by ELISA. The data are expressed as the mean ± SD for at least triplicate experiments. *p<0.05 stands for vs NC or Control-gRNA or LV-Control. #p<0.05 stands for vs NC + CCl_4.

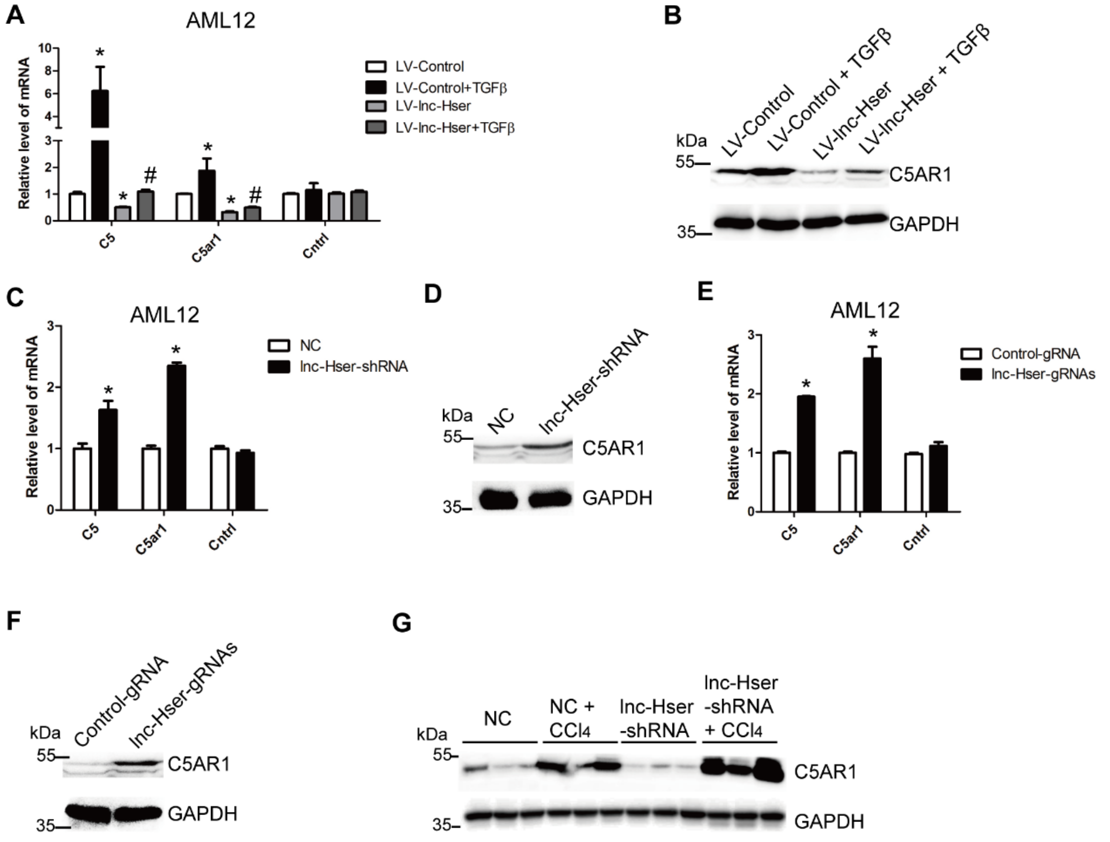

Fig. S7, related to Fig. 5. (A, B) AML12 cells were infected with LV-lnc-Hser for 72 h and further treated with 10 ng/ml TGFβ for additional 24 h. The mRNA level of *C5, C5ar1* and *Cntrl* was detected by qRT-PCR (A). The protein level of C5AR1 was detected by western blot. GAPDH was used as an internal control (B). (C-F) The expression of C5, C5AR1 and CNTRL was detected in lnc-Hser-silenced AML12 cells by qRT-PCR (C, E) and western blot. GAPDH was used as an internal control (D, F). (G) Mice were treated with oil in combination with injection of lenti-NC (Negative Control, n = 10), or CCl_4 in combination with injection of lenti-NC (NC + CCl_4, n = 10), or oil in combination with injection of lenti-lnc-Hser-shRNA (lnc-Hser-shRNA, n = 10), or CCl_4 in combination with injection of lenti-lnc-Hser-shRNA (lnc-Hser-shRNA + CCl_4, n = 10). The protein level of C5AR1 in livers of mice in each group was detected by western blot. GAPDH was used as an internal control (B). The data are expressed as the mean ± SD for at least triplicate experiments. **p*<0.05 stands for vs LV-Control or NC or Control gRNA. #*p*<0.05 stands for vs LV-Control + TGFβ.

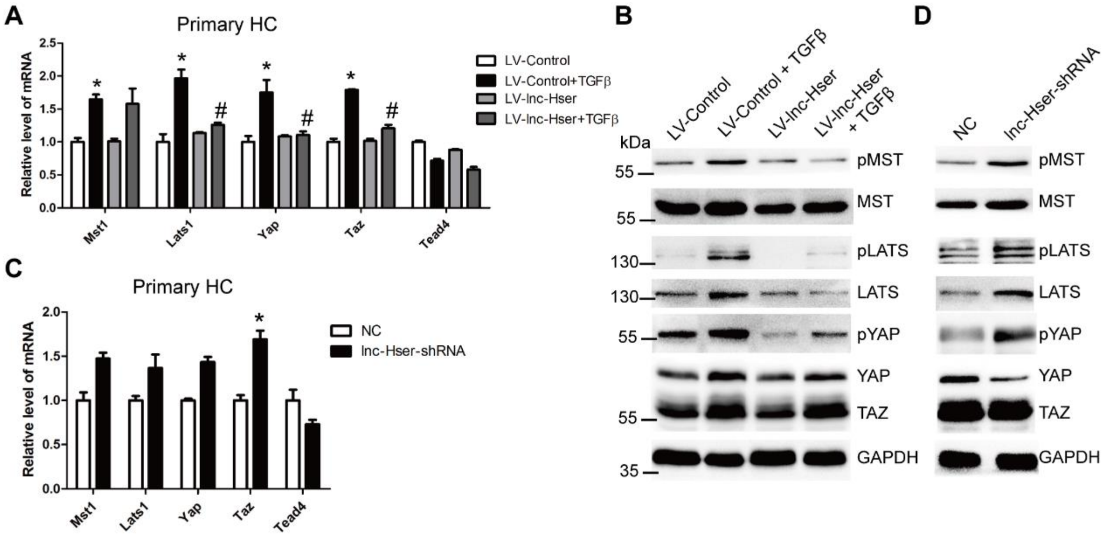

Fig. S8, related to Fig. 5. (A, B) Primary HCs were infected with LV-lnc-Hser for 72 h and further treated with 10 ng/ml TGFβ for additional 24 h. The mRNA level of *Mst1, Lats1, Yap, Taz* and *Tead4* was detected by qRT-PCR (A). The protein level of pMST, MST, pLATS, LATS, pYAP and YAP/TAZ was detected by western blot (B). GAPDH was used as an internal control. (C) The expression of *Mst1, Lats1, Yap, Taz* and *Tead4* was detected in primary HCs infected with lenti-lnc-Hser-shRNA or lenti-NC by qRT-PCR. (D) The protein level of pMST, MST, pLATS, LATS, pYAP and YAP/TAZ was detected in primary HCs infected with lenti-lnc-Hser-shRNA or lenti-NC by western blot. GAPDH was used as an internal control. The data are expressed as the mean ± SD for at least triplicate experiments. *$p<0.05$ stands for vs LV-Control or NC. #$p<0.05$ stands for vs LV-Control + TGFβ.

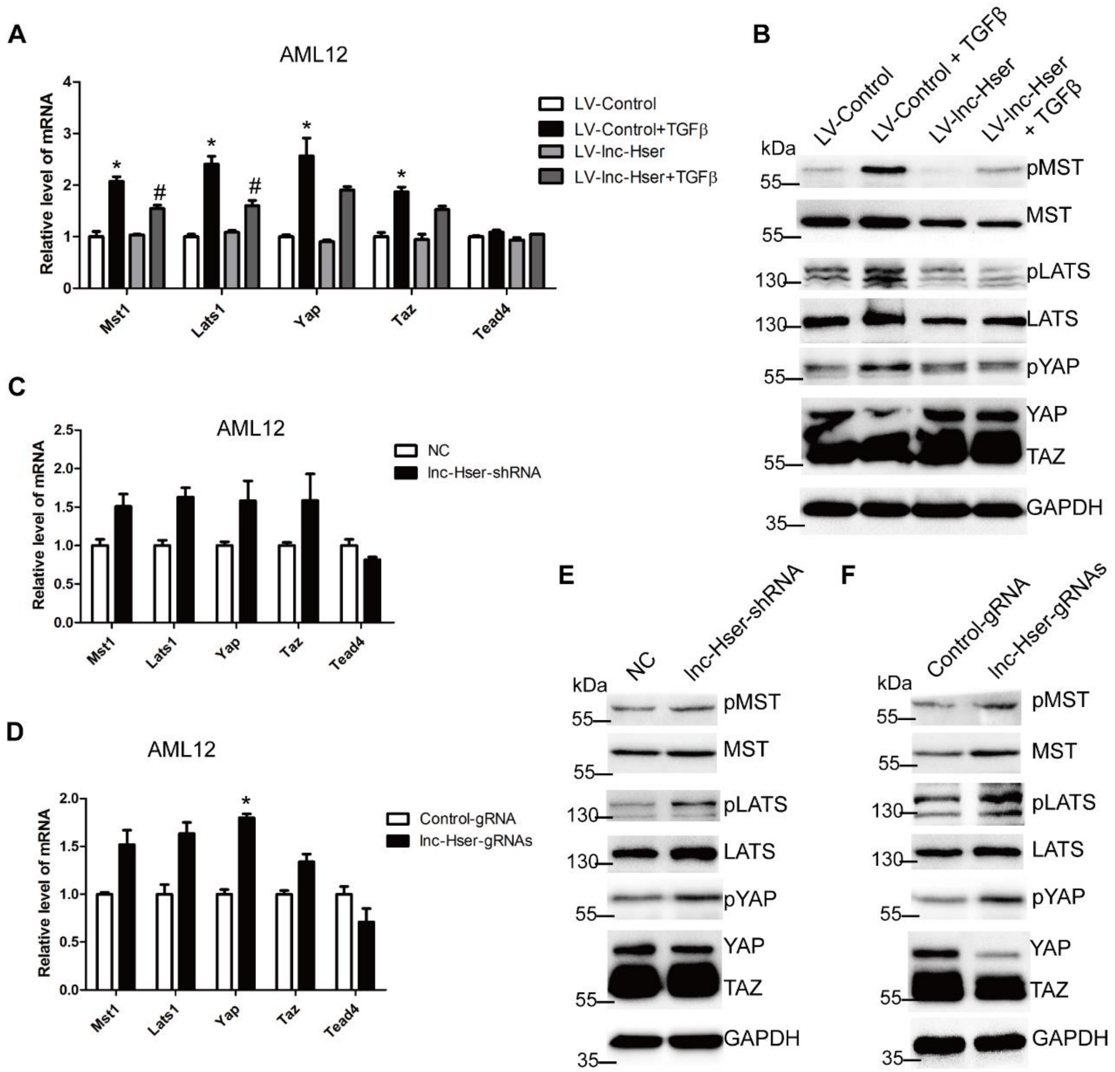

Fig. S9, related to Fig. 5. (A, B) AML12 cells were infected with LV-lnc-Hser for 72 h and further treated with 10 ng/ml TGFβ for additional 24 h. The mRNA level of *Mst1, Lats1, Yap, Taz* and *Tead4* was detected by qRT-PCR (A). The protein level of pMST, MST, pLATS, LATS, pYAP and YAP/TAZ was detected by western blot. GAPDH was used as an internal control (B). (C, D) The mRNA level of *Mst1, Lats1, Yap, Taz* and *Tead4* was detected in lnc-Hser-silenced AML12 cells by qRT-PCR. (E, F) The protein level of pMST, MST, pLATS, LATS, pYAP and YAP/TAZ was detected in lnc-Hser-silenced AML12 cells by western blot. GAPDH was used as an internal control. The data are expressed as the mean ± SD for at least triplicate experiments. **p*<0.05 stands for vs LV-Control or NC or Control-gRNA. #*p*<0.05 stands for vs LV-Control + TGFβ.

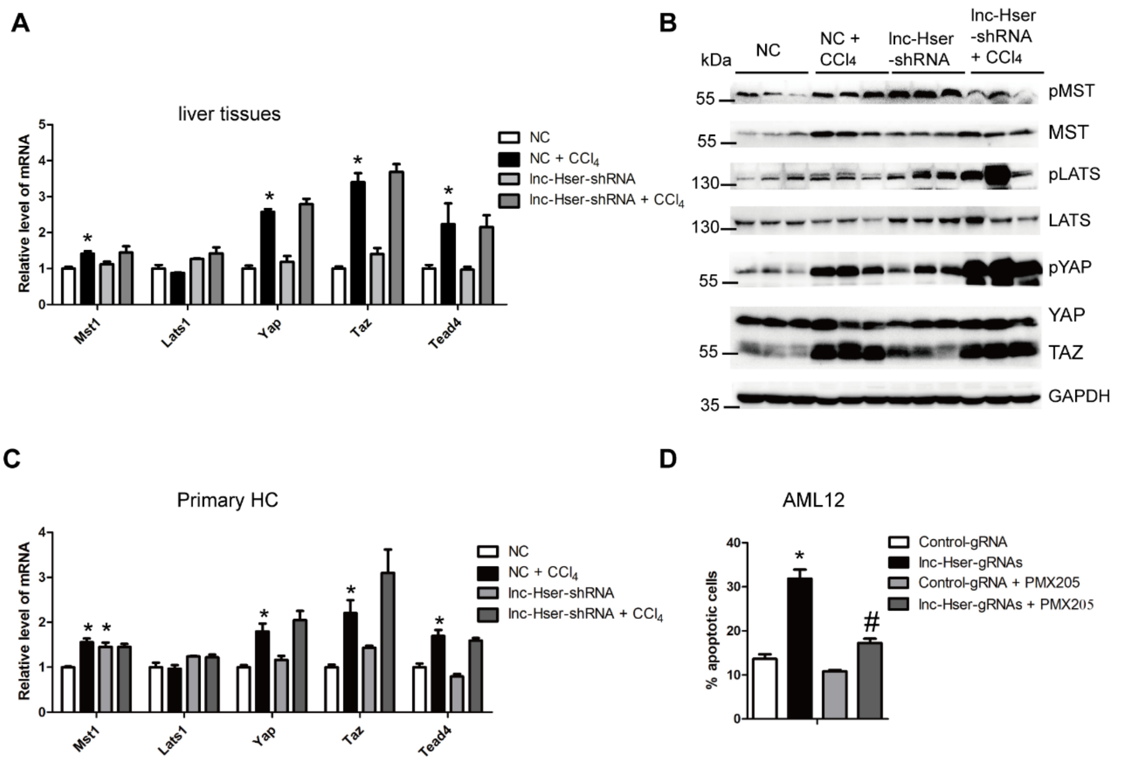

Fig. S10, related to Fig. 5. Mice were treated with oil in combination with injection of lenti-NC (Negative Control, n = 10), or CCl_4 in combination with injection of lenti-NC (NC + CCl_4, n = 10), or oil in combination with injection of lenti-lnc-Hser-shRNA (lnc-Hser-shRNA, n = 10), or CCl_4 in combination with injection of lenti-lnc-Hser-shRNA (lnc-Hser-shRNA + CCl_4, n = 10). (A) The mRNA level of *Mst1, Lats1, Yap, Taz* and *Tead4* was detected in livers by qRT-PCR. (B) The protein level of pMST, MST, pLATS, LATS, pYAP and YAP/TAZ was detected in livers by western blot. GAPDH was used as an internal control. (C) The mRNA level of *Mst1, Lats1, Yap, Taz* and *Tead4* was detected in the primary HCs isolated from mice in each group, by qRT-PCR. (D) The expression of lnc-Hser was stably knocked down by the CRISPR/Cas9 system with guide RNA pairs in AML12 cells. PMX205, a specific inhibitor of C5AR1, was added in lnc-Hser-silenced AML12 cells for 24 h. Cell apoptosis was determined by FACS analysis. The data are expressed as the mean ± SD for at least triplicate experiments. *p<0.05 stands for vs NC or Control-gRNA. #p<0.05 stands for vs NC + CCl_4 or lnc-Hser-gRNAs.

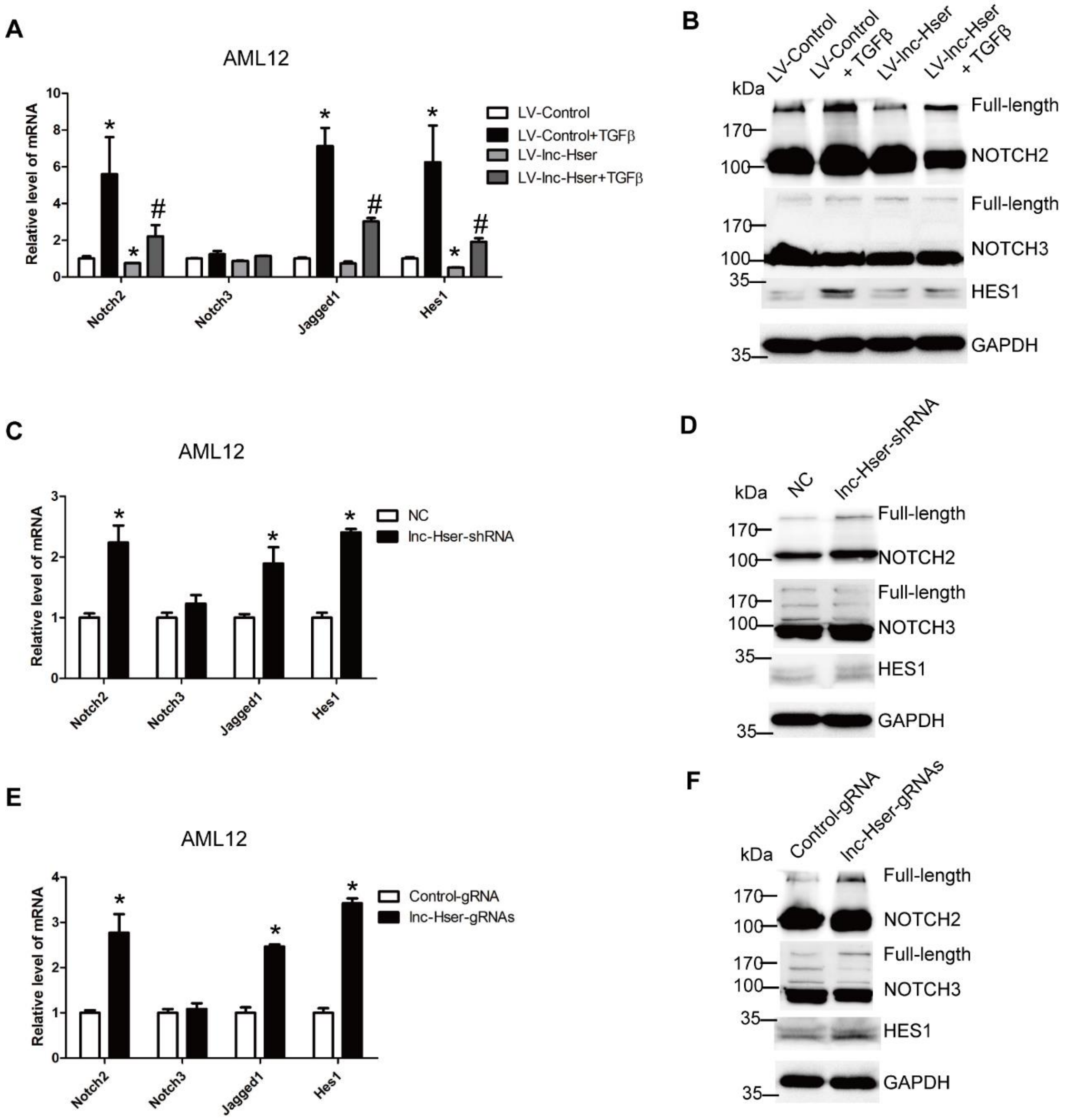

Fig. S11, related to Fig. 6. (A, B) AML12 cells were infected with LV-lnc-Hser for 72 h and further treated with TGFβ for additional 24 h. The expression of Notch2, Notch3, Jagged1 and Hes1 was detected by qRT-PCR (A) and western blot. GAPDH was used as an internal control (B). (C, D) The expression of Notch2, Notch3, Jagged1 and Hes1 in AML12 cells infected with lenti-lnc-Hser-shRNA or lenti-NC was detected by qRT-PCR (C) and western blot. GAPDH was used as an internal control (D). (E, F) The expression of Notch2, Notch3, Jagged1 and Hes1 in lnc-Hser-silenced AML12 cells was detected by qRT-PCR (E) and western blot. GAPDH was used as an internal control (F). The data are expressed as the mean ± SD for at least triplicate experiments. *$p<0.05$ stands for vs LV-Control or NC or Control gRNA. #$p<0.05$ stands for vs LV-Control + TGFβ.

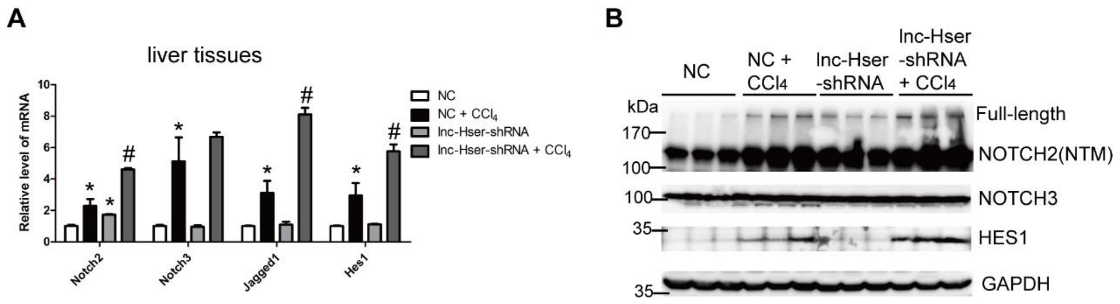

Fig. S12, related to Fig. 6. Mice were treated with oil in combination with injection of lenti-NC (Negative Control, n = 10), or CCl_4 in combination with injection of lenti-NC (NC + CCl_4, n = 10), or oil in combination with injection of lenti-lnc-Hser-shRNA (lnc-Hser-shRNA, n = 10), or CCl_4 in combination with injection of lenti-lnc-Hser-shRNA (lnc-Hser-shRNA + CCl_4, n = 10).(A) The mRNA level of *Notch2, Notch3, Jagged1* and *Hes1* was detected in livers by qRT-PCR. (B) The protein level of Notch2, Notch3 and Hes1 was detected in livers by western blot. GAPDH was used as an internal control. The data are expressed as the mean ± SD for at least triplicate experiments. *$p<0.05$ stands for vs NC. #$p<0.05$ stands for vs NC + CCl_4.

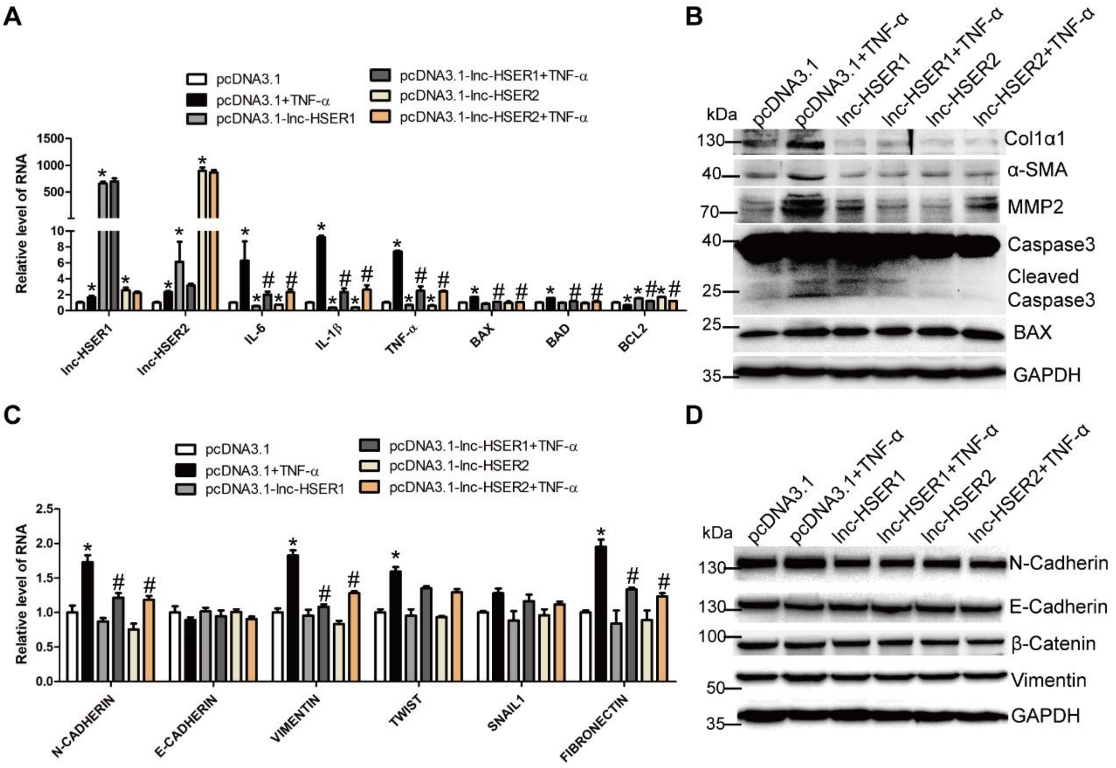

Fig. S13, related to Fig. 7. lnc-HSER1/2 ameliorates TNF-α-induced apoptosis and inflammation of L02 cells. (A-D) L02 cells were transfected with pcDNA3.1, pcDNA3.1-lnc-HSER1 and pcDNA3.1-lnc-HSER2 for 48 h and further treated with TNF-α for additional 24 h. (A) The RNA level of *lnc-HSER1, lnc-HSER2,* pro-inflammation genes, apoptosis-related genes was detected by qRT-PCR. (B) The protein level of α-SMA, Col1α1, MMP2, total and cleaved Caspase3 and BAX was detected by western blot. GAPDH was used as an internal control. (C, D) The expression of EMT-related genes was detected by qRT-PCR (C) and western blot (D). GAPDH was used as an internal control. The data are expressed as the mean ± SD for at least triplicate experiments. **p*<0.05 stands for vs pcDNA3.1. #*p*<0.05 stands for vs pcDNA3.1 + TNF-α.

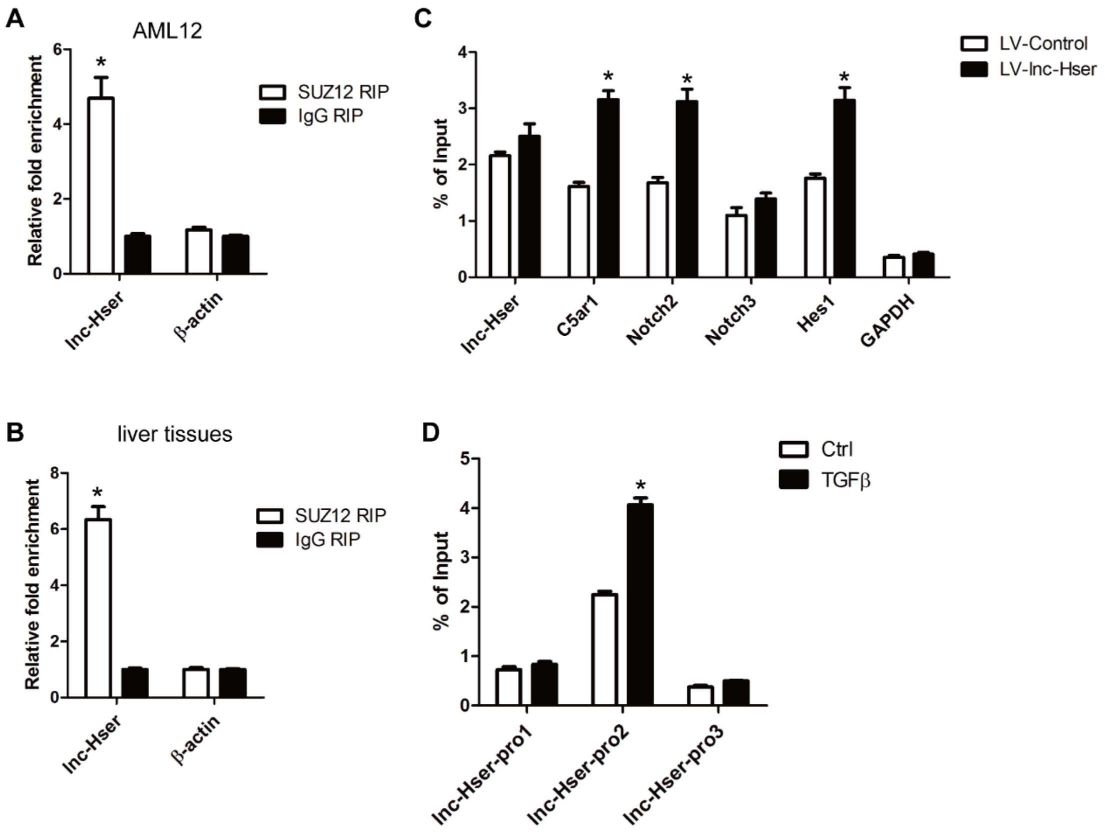

Fig. S14. lnc-Hser interacts with PRC2. (A, B) qRT-PCR detection of lnc-Hser and β-Actin retrieved by SUZ12-specific antibody compared with IgG in the RIP assay with AML12 cells (A) and the single cell suspensions isolated from mouse liver (B). (C) AML12 cells were infected with LV-lnc-Hser or LV-Control, and ChIP analyses were performed on indicated genes promoter regions using anti-SUZ12 antibody. (D) AML12 cells were treated with or without TGFβ, and ChIP analyses were performed on the promoter regions of lnc-Hser using anti-SUZ12 antibody. Enrichment was shown relative to input. The data are expressed as the mean ± SD for at least triplicate experiments, *p<0.05.

Supplementary Tables

Table S1. Primers and Oligonucleotides

qRT-PCR primers for analysis of transcript levels

Gene symbol	Forward 5' - 3'	Reverse 5 '- 3'
Mouse lnc-Hser	GCTCTTTCATGGGAGCAACT	TCATTGCCTTTGGCTTTCTC
Mouse lnc-Hser	GCCAAAGGCAATGAGACTCC	CAAATGCCTCAGTATGGCCG
Mouse β-Actin	ATGCCACAGGATTCCATACCCAAGA	CTCTAGACTTCGAGCAGGAGATGG
Mouse Gapdh	GGCATGGACTGTGGTCATGAG	TGCACCACCAACTGCTTAGC
Mouse Malat1	AAATTGATGGCCTTTTCTGG	AGCTGGATCCTTGAGGTCAC
Mouse Col1α1	ATCGGTCATGCTCTCTCCAAACCA	ACTGCAACATGGAGACAGGTCAGA
Mouse Col1α2	CCTTTGTCAGAATACTGAGCAGC	GTAACTTCGTGCCTAGCAACA
Mouse Col3α1	TGCTCCAGTTAGCCCTGCAA	GGTCCTGCAGGCAACAGTGGTTC
Mouse Col4α5	CTCCCTTACCGCCCTTTTCTC	AGGCGAAATGGGTATGATGGG
Mouse Acta2	TCGGATACTTCAGCGTCAGGA	GTCCCAGACATCAGGGAGTAA
Mouse Pcna	TTTGAGGCACGCCTGATCC	GGAGACGTGAGACGAGTCCAT
Mouse Ki67	CATCCATCAGCCGGAGTCA	TGTTTCGCAACTTTCGTTTGTG
Mouse Ctgf	ATCCAGGCAAGTGCATTGGTA	GGGCCTCTTCTGCGATTTC
Mouse Bax	TTGCTGATGGCAACTTCAAC	GATCAGCTCGGGCACTTTAG
Mouse Bad	AGAGTATGTTCCAGATCCCAG	GTCCTCGAAAAGGGCTAAGC
Mouse Bcl2	GCTGGGATGCCTTTGTGGAACT	CAGAGACAGCCAGGAGAAATCAAAC
Mouse Tnfα	CATCTTCTCAAAATTCGAGTGACAA	TGGGAGTAGACAAGGTACAACCC
Mouse Il-1β	GTCGCTCAGGGTCACAAGAA	GTGCTGCCTAATGTCCCCTT
Mouse Mcp1	GTTAACGCCCCACTCACCTG	GGGCCGGGGTATGTAACTCA
Mouse Il-6	AGTTGCCTTCTTGGGACTGA	TCCACGATTTCCCAGAGAAC
Mouse Mmp2	GTGTTCTTCGCAGGGAATGAG	GATGCTTCCAAACTTCACGCT
Mouse Mmp9	ACCACAGCCAACTATGACCAGGAT	AAGAGTACTGCTTGCCCAGGAAGA
Mouse Tgfβ1	TGTGTTGGTTGTAGAGGGCAAGGA	TTTGGAGCCTGGACACACAGTACA
Mouse Pdgfβ1	CTGCCACAGCATGATGAGGAT	GCCAGGATGGCTGAGATCACCAC
Mouse E-Cadherin	AACCCAAGCACGTATCAGGG	GAGTGTTGGGGGCATCATCA

Mouse N-Cadherin	ACAGCGCAGTCTTACCGAAG	TGGCTCGCTGCTTTCATAC
Mouse Vimentin	CTTGAACGGAAAGTGGAATCCT	GTCAGGCTTGGAAACGTCC
Mouse Fibronectin	GCTCAGCAAATCGTGCAGC	CTAGGTAGGTCCGTTCCCACT
Mouse Snail1	CACACGCTGCCTTGTGTCT	GGTCAGCAAAAGCACGGTT
Mouse Twist	CTGCCCTCGGACAAGCTGAG	CTAGTGGGACGCGGACATGG
Mouse Notch2	TGACTGTTCCCTCACTATGG	CACGTCTTGCTATTCCTCTG
Mouse Notch3	TTGTCTGGATGGAAGCCCATGT	ACTGAACTCTGGCAAACGCCT
Mouse Jagged1	GGGAGAGTGATACTTGATGGG	CTCATTGTGGCTTTTGTGGAG
Mouse Hes1	CTCCCGGCATTCCAAGCTAG	AGCGGGTCACCTCGTTCATG
Mouse Mst1	GAACACAGACCTGTGGATTG	CGCCTTGATATCTCGGTGTA
Mouse Mst2	TCTCCTCAATACAGAAGGAC	AGAAGTAATGCCAAGGGACC
Mouse Lats1	TGGTGACTCTGGGGATAAAGAA	GGGAGTAACTCTGAATCCGAGAC
Mouse Lats2	ATCCTCCCAAAGGGTACAGCACAG	TGGTGGCGTCTTGTTCTGGAAG
Mouse Yap	ACCCTCGTTTTGCCATGAAC	CCTTCTCCATCTGTAACTGC
Mouse Taz	TCCTATGACGTGACCGACGA	GGGTCTTGCCATGTGGTGAT
Mouse Tead4	TGATGCAGAGGGTGTATGGA	GATCAGCTCATTCCGACCAT
Mouse C5	CCTGCTGAAGCCCAAGAGAA	GCAGGGTGTTTTCAAGCAGG
Mouse C5ar1	AGGTCTCTCCCCAGCATCAT	GTCGTGGACGGAGTGAAAGT
Mouse Cntrl	AGAAGCGTGAAGATGCCAGA	GCTGGTCTTTGGCAATGGTG
Human β-ACTIN	GCCGGGACCTGACTGACTAC	TTCTCCTTAATGTCACGCACGAT
Human GAPDH	ACCCAGAAGACTGTGGATGG	TTCAGCTCAGGGATGACCTT
Human lnc-HSER1	AGTAGTCACTGAGGCTGACG	GCCTCTCAGCGTACTTCCG
Human lnc-HSER2	CGTGCAAGTGTGTAGAAGCTG	ATGAACGAATGAATTCTCACCAG
ENST00000466280	AGGCGTTGGCATTTCAAACA	TGGATCTGTTCTCCTCGTACA
ENST00000489802	CTGCGTATGCTCTTTCCCTG	ACATAGGATACTCAATGCATATT
NR_148450	CTGGGGCCGAAAGAACAGTC	TGCCTGTCTTTGTGTGGTTGA
Human COL1α1	AACCAAGGCTGCAACCTGGA	GGCTGAGTAGGGTACACGCAGG
Human ACTA2	GCCATGTTCTATCGGGTACTTC	CAGGGCTGTTTTCCCATCCAT
Human IL-6	CAGGAGCCCAGCTATGAACT	GAAGGCAGCAGGCAACAC

Human IL-1β	GCAGAAGTACCTGAGCTCGC	CTTGCTGTAGTGGTGGTCGG
Human TNF-α	CCTGCCCCAATCCCTTTATT	CCCTAAGCCCCCAATTCTCT
Human BAX	TCAGGATGCGTCCACCAAGAA	TCTGCAGCTCCATGTTACTGTCCA
Human BAD	CAGACCCGGCAGACAGATGAG	CTCACTCGGCTCAAACTCTGG
Human BCL2	GTGGAGAGCGTCAACCGGGAGA	GGGCCGTACAGTTCCACAAAGGC
Human N-CADHERIN	TCAGGCTCCAAGCACCCCTTCA	ATGACGGCCGTGGCTGTGTT
Human E-CADHERIN	CATGAGTGTCCCCCGGTATC	CAGTATCAGCCGCTTTCAGA
Human VIMENTIN	ATTCCACTTTGCGTTCAAGG	CTTCAGAGAGAGGAAGCCGA
Human TWIST	TGCGGAAGATCATCCCCACG	GCTGCAGCTTGCCATCTTGGA
Human SNAIL1	GCACATCCGAAGCCACAC	GGAGAAGGTCCGAGCACAC
Human FIBRONECTIN	CTTTGGTGCAGCACAACTTC	TCCTCCTCGAGTCTGAACCA
Human C5AR1	TCCTGCCCTCCCTCATC	GCTGTAGTCCACGCCAC
Human C5	ATGAAACCTGTGAGCAGCGA	GCTTGCGACGACACAACATT

Cloning primers for lnc-Hser

Name	Sequence 5' - 3'
lnc-Hser 5' BamHI F1	cgcggatccGGTTGCTGTTTGTTAGCAGGC
lnc-Hser 5' BamHI R1	cgcggatccTATGATTGCAGTGTTTTGGA
lnc-HSER1 5' BamHI F1	cgcggatccCTTCAAGATTCCGTCTAATCC
lnc-HSER1 5' BamHI R1	cgcggatccAGTGTCAAAAGTAAAGTAGATAAC
lnc-HSER1 5' BamHI F2	cgcggatccCTTCAAGATTCCGTCTAATC
lnc-HSER1 5' Xho1 R2	ccgctcgagAGTGTCAAAAGTAAAGTAGA
lnc-HSER2 5' BamHI F1	cgcggatccCCTGCCACTTTCACAGTGTAC
lnc-HSER2 5' BamHI R1	cgcggatccCCCCCAAAACATGCAATTTACC
lnc-HSER2 5' BamHI F2	cgcggatccCCTGCCACTTTCACAGTGTA
lnc-HSER2 5' Xho1 R2	ccgctcgagCCAAAACATGCAATTTACCC

shRNA sequences

Name	Sequence 5' - 3'
Mouse sh-lnc-Hser-1 Forward	GATCCCCGGACTGTATTTGTCACAAGTTCAAGAGACTTGTGACAAATACAGTCCTTTTTA
Mouse sh-lnc-Hser-1 Reverse	AGCTTAAAAAGGACTGTATTTGTCACAAGTCTCTTGAACTTGTGACAAATACAGTCCGGG
Mouse sh-lnc-Hser-2 Forward	GATCCCCCCATGGCTGAGTCCTCATTTTCAAGAGAAATGAGGACTCAGCCATGGTTTTTA
Mouse sh-lnc-Hser-2 Reverse	AGCTTAAAAACCATGGCTGAGTCCTCATTTCTCTTGAAAATGAGGACTCAGCCATGGGGG
Negative control Forward	GATCCCCGTTCTCCGAACGTGTCACGTTCAAGAGACGTGACACGTTCGGAGAACTTTTTA
Negative control Reverse	AGCTTAAAAAGTTCTCCGAACGTGTCACGTCTCTTGAACGTGACACGTTCGGAGAACGGG

siRNA sequences

Name	Forward 5' - 3'	Reverse 5' - 3'
Mouse si-lnc-Hser-1	GGACUGUAUUUGUCACAAGTT	CUUGUGACAAAUACAGUCCTT
Mouse si-lnc-Hser-2	CCAUGGCUGAGUCCUCAUUTT	AAUGAGGACUCAGCCAUGGTT
negative control	GUUCUCCGAACGUGUCACGTT	CGUGACACGUUCGGAGAACTT

gRNA sequences

Name	Sequence 5' - 3'
Mouse gRNA1 Forward	caccgATATGCTCCCGGACCCACCC
Mouse gRNA1 Reverse	aaacGGGTGGGTCCGGGAGCATATc
Mouse gRNA2 Forward	caccgCGGCTCACCTGTAAGTTACG
Mouse gRNA2 Reverse	aaacCGTAACTTACAGGTGAGCCGc

RACE primers for Mouse-lnc-Hser

gene specific primer	Sequence 5' - 3'
3' OUTER PRIMER	GATTACGCCAAGCTTGCTGAGCAGTGCCAGAGCATCAGAGCCA
5' OUTER PRIMER	GATTACGCCAAGCTTGCCATGGGAGCCTGGCCCTGTGAAGA
5' INNER PRIMER	GATTACGCCAAGCTTGCAGGAGTCTCATTGCCTTTGGCTTTC

Primers for Mouse ChIP qRT-PCR

Locus	Forward 5' - 3'	Reverse 5 '- 3'
lnc-Hser (-136--18)	TTTGCCTCGTGCTCT	CCTCACATTTATGTACCCT
lnc-Hser (-913--806)	TAAGCGCATTGCCCCTTCTC	TGGTAGCATCCTTGGTCCTG
lnc-Hser (-1994--1819)	ATGATGCCAAAAGCCCTCTGT	AGGGCAGAGCAGAAGTTGAT
C5aR (-131—36)	CACATCTCCCTAACCCCCTT	CCCAGCCTGGTGGCTTTTAT
C5aR (-800--711)	ACAGAGTGTTGGGATTGCGT	CCACCTGCATAGGAAGGACC
C5aR (-1906--1827)	GAACCTCTTGCAAGCCCACC	GAACCTCTTGCAAGCCCACC
Notch2 (-371--300)	TTTGATGTTGGGCGCTTCAG	GGTTTCCCGCAGAAAGAAGC
Notch2 (-1123--1014)	CACCCATTTGCACTTGCTGAA	ACACGGGGAAGTCTTTATGGC
Notch2 (-1965-1890)	GGTAACACCATGGGTGAACAAA	GGCAATTTCTGCTTGTGCCAT
Notch3 (-234--165)	TTGCAGACCTCGGTACACTC	GATACCTGTCACGTCACGCA
Notch3 (-1083--1014)	CTCCATCACTAGGAGACCAAAGG	GTGTCTGTGTATGCCCTTCCA
Notch3 (-1908--1765)	AGAACCTGGGGTTTCCAGTG	GGGATCCAGTCTTCGGTCCA
Hes1 (-223--60)	TTGACGTTGTAGCCTCCGGT	AACGGCTCGTGTGAAACTTCC
Hes1 (-1199-1007)	CAGCTGCTATTTACCTTCTTGGC	AGCACGTGCCAGGATGTTTT
Hes1 (-1609--1522)	AAGTGCGGTCAGGCATCTC	ATCTGAGCGTGGCCGAAAC
Gapdh intron	ATCCTGTAGGCCAGGTGATG	AGGCTCAAGGGCTTTTAAGG

Table S2. Baseline characteristics of patients with liver tissue

Metavir score	Healthy (F0)	Mild fibrosis (F1-F2)	Advanced fibrosis (F3-F4)
Cases(n)	6	16	12
Age (years)*	57.7 ±15.7	53.6 ±10.3	50.8 ± 9.4
Male sex (*n* (%))	4 (66.7)	7 (43.8)	7(58.3)
ALT (U/L)*	22.0 ± 12.3	34.7 ± 32.0	32.5 ± 15.2
AST (U/L)*	26.5 ± 12.6	60.5 ± 54.2	39.1 ± 16.3
ALB (g/L)*	38.3 ± 7.9	41.3 ± 3.7	43.9 ±12.1
GGT (U/L)*	47.3 ± 25.0	72.9 ± 50.4	122.5 ± 89.2
Etiology (*n* (%))			
Biliary Obstruction	0 (0)	2 (12.5)	0 (0)
HBV	0 (0)	14 (87.5)	11 (91.7)
HCV	0 (0)	0 (0)	1 (8.3)

*Mean ± SD.

ALT, alanine aminotransferase; AST, aspartate aminotransferase; ALB, Albumin; GGT, γ-glutamyl transpeptadase; HBV, hepatitis B virus; HCV, hepatitis C virus.

Table S3. Serum levels of ALT, AST in CCl_4-induced liver fibrosis model (mean ± SD, n = 5)

Group	ALT (U/L)	AST (U/L)
NC group	38.8 ± 8.4	47.9 ± 5.3
NC + CCl_4 group	157.5 ± 23.7[*]	205.3 ± 35.8[*]
lnc-Hser-shRNA group	44.9 ± 5.5	53.4 ± 10.5
lnc-Hser-shRNA + CCl_4 group	261.6 ± 67.8[#]	293.9 ± 46.5[#]

[*]p<0.05 compared with the NC group. [#]p<0.05 compared with NC + CCl_4 group. All statistical analyses were performed using SPSS version 13.0 software and p<0.05 indicated statistical significance.
